# Mechanism of retinoic acid-induced transcription: histone code, DNA oxidation and formation of chromatin loops

**DOI:** 10.1093/nar/gku823

**Published:** 2014-09-12

**Authors:** Candida Zuchegna, Fabiana Aceto, Alessandra Bertoni, Antonella Romano, Bruno Perillo, Paolo Laccetti, Max E. Gottesman, Enrico V. Avvedimento, Antonio Porcellini

**Affiliations:** 1 Dipartimento di Biologia, Università Federico II, 80126 Napoli, Italy; 2 Dipartimento di Medicina e di Scienze della Salute, Università del Molise, 86100 Campobasso, Italy; 3 Dipartimento di Medicina Molecolare e Biotecnologie mediche, Istituto di Endocrinologia ed Oncologia Sperimentale del C.N.R., Università Federico II, 80131 Napoli, Italy; 4 Istituto di Scienze dell'Alimentazione, C.N.R., 83100 Avellino, Italy; 5 Institute of Cancer Research, Columbia University Medical Center, New York, NY 10032, USA

## Abstract

Histone methylation changes and formation of chromatin loops involving enhancers, promoters and 3′ end regions of genes have been variously associated with active transcription in eukaryotes. We have studied the effect of activation of the retinoic A receptor, at the RARE–promoter chromatin of *CASP9* and *CYP26A1* genes, 15 and 45 min following RA exposure, and we found that histone H3 lysines 4 and 9 are demethylated by the lysine-specific demethylase, LSD1 and by the JMJ-domain containing demethylase, D2A. The action of the oxidase (LSD1) and a dioxygenase (JMJD2A) in the presence of Fe++ elicits an oxidation wave that locally modifies the DNA and recruits the enzymes involved in base and nucleotide excision repair (BER and NER). These events are essential for the formation of chromatin loop(s) that juxtapose the RARE element with the 5′ transcription start site and the 3′ end of the genes. The RARE bound-receptor governs the 5′ and 3′ end selection and directs the productive transcription cycle of RNA polymerase. These data mechanistically link chromatin loops, histone methylation changes and localized DNA repair with transcription.

## INTRODUCTION

Retinoic acid (RA), an active derivative of vitamin A, plays a role in the regulation of embryonic development, homeostasis and differentiation of adult tissues. RA metabolites, collectively known as retinoids, are well-characterized inhibitors of cancer cell proliferation or inducers of stem cell differentiation. The biological activity of RA is mediated by its binding to RA receptors (RARα, RARβ and RARγ) that function as hetero-dimers with retinoid X receptors (RXRs), targeting DNA at specific sites, known as RA responsive elements (RAREs). Following hormone binding, an active receptor complex induces covalent modifications at the N-terminal tails of nucleosomal histones and assembles an active transcription complex on chromatin (1).

Despite extensive studies on RA-induced transcription, it is not known if there is a common set of histone modifications or how the initiation transcription complex is assembled on regulatory regions. The H3 methylation changes reported so far associated with activation of the receptor(s) by RA, may be secondary to repression of transcription (2) or induced by cell differentiation (1,3). Although large DNA domains and histone modifications have been studied during development, the mechanism used by RA to activate transcription still remains elusive.

To address this issue, we studied two prototypic genes induced by RA: caspase 9 (*CASP9*) and Cyp26A1 (*CYP26A1*). *CASP9* contains a functional RARE located 9.5 kb downstream of the transcription start site (TSS) (4), whereas the RA-induced *CYP26A1* expression is driven by a compact RARE–promoter (5). We first analyzed the recruitment of RA receptors and RNA polymerase II to the promoter and RARE sites. Second, we studied the changes of methylation of lysine 4 (K4) and lysine 9 (K9), following the recruitment on the chromatin sites of two demethylating enzymes, LSD1 (KDM1A) and JMJD2A (KDM4A). It has been reported that LSD1 demethylates H3K4me2 or H3K9me2 and JMJD2A demethylates H3K4-K9 me3 (6). Third, we analyzed the formation after RA exposure of specific chromatin-DNA domains that connect the 5′ end-promoter-RARE and the 3′ end site of the RA-target gene. Our data indicate that histone demethylation, DNA oxidation and chromatin looping induced by RA are temporally and causally associated with the onset of productive transcription induced by RA, and inhibition of any of these three events abolishes RA-induced transcription.

## MATERIALS AND METHODS

### Cells and transfections

Human breast cancer MCF-7 cells were grown at 37°C in 5% CO_2_ in Dulbecco's modified Eagle's medium (DMEM) supplemented with phenol red, L-glutamine (2 mM), insulin (10 μg/ml), hydrocortisone (3.75 ng/ml) and 10% fetal bovine serum (FBS) (Invitrogen). Non-tumorigenic breast MCF10-2A cells were grown in a 1:1 mixture of DMEM and Ham's F12 medium supplemented with 20-ng/ml epidermal growth factor, 100-ng/ml cholera toxin, 0.01-mg/ml insulin and 500-ng/ml hydrocortisone, 95%; horse serum, 5%. Cells were provided with fresh medium every 3 days. To evaluate the effect of RA, cells were grown in phenol red-free medium containing 10% dextran-charcoal-stripped FBS for 1–3 days, before being challenged with 300-nM RA for different times according to the experimental protocol.

To obtain *LSD1* knock down with siRNA, cells were transiently transfected, using a Neon^®^ Transfection System, with siRNA in medium without serum to a final concentration of 10 nM and incubation was continued for 48 h. Scrambled RNA, at the same concentration, was used as negative control. The same procedure was used to obtain *JMJD2A, OGG1* and *APE1* knock down with the specific siRNAs (see Supplemental Information). To rescue LSD1 activity in knock down experiments with siRNAs, LSD1 full-length cDNA was inserted into the CMV 3xFLAG expression vector (Sigma-Aldrich). LSD1ALA mutant has been described elsewhere (7,8). To rescue JMJD2A activity, cells were transfected with pCMV6-AC-GFP plasmid containing JMJD2A full-length (RG200574, OriGene Technologies, Inc.). To assess the transfection efficiency at single cell level, all transfections were traced with pEGFP Vector (Clontech) or with BLOCK-iT Alexa Fluor^®^ Red Fluorescent Control and analyzed by fluorescence-activated cell sorting (FACS).

### RNA extraction and quantitative reverse transcription polymerase chain reaction and quantitative polymerase chain reaction

Total RNA was extracted using TRIzol (Gibco/Invitrogen). cDNA was synthesized in a 20-μl reaction containing 1 μg of total RNA, 100 U of Superscript III Reverse Transcriptase (Invitrogen) and 2 μl random hexamer (20 ng/μl) (Invitrogen). mRNA was reverse-transcribed for 1 h at 50°C, and the reaction was heat inactivated for 15 min at 70°C. The products were stored at −20°C. Quantitative reverse transcription polymerase chain reactions (PCRs) and quantitative PCRs (qPCRs) were performed on a 7500 Real Time PCR System (Applied Biosystems) using the SYBR Green-detection system (FS Universal SYBR Green MasterRox/Roche Applied Science). The complete list of oligonucleotides used is reported in Supplementary Table S1.

### Chromatin immunoprecipitation

Cells were transfected and/or treated as indicated in the legends of the figures. The cells (∼2.5 x 10^6^ for each antibody) were fixed for 10 min at room temperature by adding 1 volume of 2% formaldehyde to a final concentration of 1%; the reaction was quenched by addition of glycine to a final concentration of 125 mM. Fixed cells were harvested and the pellet was resuspended in 1 ml of Lysis Buffer (see Supplemental Information) containing 1X protease inhibitor cocktail (Roche Applied Science). The lysates were sonicated to have DNA fragments 300–600 bp. Sonicated samples were centrifuged and supernatants diluted 2-fold in the chromatin immunoprecipitation (ChIP) buffer (Supplemental Information). An aliquot (1/10) of sheared chromatin was further treated with proteinase K, extracted with phenol/chloroform and precipitated to determine DNA concentration and shearing efficiency (input DNA). The ChIP reaction was set up according to the manufacturer's instructions. Briefly, the sheared chromatin was precleared for 2 h with 1 μg of non-immune IgG (Santa Cruz Biotechnology) and 20 μl of Protein A/G PLUS-Agarose (Santa Cruz Biotechnology) saturated with salmon sperm (1 mg/ml). Precleared chromatin was divided in aliquots and incubated at 4°C for 16 h with 1 μg of the specific antibody (Supplemental Information) and non-immune IgG, respectively. The immunocomplexes were recovered by incubation for 3 h at 4°C with 20 μl of protein-A/G agarose, beads were washed with wash buffers according to the manufacturer's instructions and immunoprecipitated DNA was recovered and subjected to qPCR using the primers indicated in the legend of the specific figures, primers sequences and qPCR protocols are described in Supplemental Information and in Supplementary Table S1.

### 8-Oxo-7, 8-dihydro-2′-deoxyguanosine DNA assay

For 8-Oxo-7, 8-dihydro-2′-deoxyguanosine (8-oxo-dG) detection, 10^6^ MCF-7 cells were seeded onto glass slides and treated with 200 or 500-nM RA for 15–30 min. Control cultures were treated with equivalent vehicle volumes and concentrations. After treatments, the cells were fixed 15 min with 4% paraformaldehyde in phosphate buffered saline (PBS). The slides were then washed three times with Tris-buffered saline (TBS)/Tween-20 and permeabilized by serial washes in methanol solutions, prior to be washed with TBS/Tween-20, blocked for 1 h at 37°C and incubated with fluorescein isothiocyanate-labeled protein, that binds 8-oxo-dG, for 15 h at 4°C (Biotrin OxyDNA Test, Biotrin, UK). Cover slips were mounted in Moviol and viewed by fluorescence. To obtain LSD1 knock down, cells were transfected with specific or control siRNAs. After 48 h, cells were subjected to different treatments, according to experimental needs, and processed for fluorescence microscopy. For single-cell transfection assays, cells were co-transfected with BLOCK-iT Alexa Fluor^®^ Red Fluorescent Control. The efficiency of transfection was 65%+10. All images were captured with Axiocam microscopy (Zeiss) with a 63x objective in the same conditions of brightness and contrast.

### Chromosome conformation capture

The chromosome conformation capture (3C) assay was performed as described (9) with minor adaptations. Briefly, the NcoI restriction enzyme was used. The cells (2.5 × 10^6^) were crosslinked in 12 ml of PBS with 1% formaldehyde for 10 min at room temperature. The reaction was quenched by addition of glycine to a final concentration of 125 mM. Fixed cells were harvested and the pellet resuspended in 1 ml of ice-cold lysis buffer (the same used for ChIP experiments). Nuclei were washed with 0.5 ml of restriction enzyme buffer, centrifuged and resuspended in 100 μl of restriction enzyme buffer. Sodium dodecyl sulphate (SDS) was added to a final concentration of 0.1%, and nuclei were incubated at 37°C for 15 min. Triton X-100 was added to a final concentration of 1% to sequester SDS. Digestion was performed with 100 U of restriction enzyme at 37°C for 16 h. The restriction enzyme was inactivated by addition of SDS to 2% and incubation at 65°C for 30 min. The reaction was diluted into 1-ml ligation reaction buffer (see Supplemental Methods) and incubated at 16°C for 18 h with 50 U of T4 DNA Ligase (Roche Applied Science). Ethylenediaminetetraacetic acid (10 mM) was added to stop the reactions. Samples were treated with Proteinase K (200 μg/ml) and incubated for 5 h at 55°C and then overnight at 65°C to reverse the formaldehyde crosslinks. The following day, the DNA was purified by phenol/chloroform extraction and ethanol precipitation. Samples were redissolved in 20 μl of Tris-EDTA (TE) buffer. To prepare a control template, we used a pool of plasmids containing an equimolar amount of the *CASP9* or *CYP26A1* inserts spanning the genomic regions of interest. Five micrograms of plasmid DNA were digested with NcoI in 50 μl of 1x buffer for 8 h at 37°C and then ligated in 20 μl with 5 U of T4 Ligase at 16°C for 4 h. The efficiency of digestion at the end of 3C treatment was quantified by real-time PCR, amplifying a fragment spanning two NcoI (uncut) in different 3C DNA preparations. Primer sequences and positions and details of PCR and qPCR protocols are described in Supplemental Information and Supplementary Table S1. PCR products were separated on 1.2% agarose gels, stained with ethidium bromide and quantified with the imageJ program (Rasband WS, ImageJ, National Institutes of Health, Bethesda, MD, USA; http://rsb.info.nih.gov/ij/). The amplified fragments at the end of the procedure were verified by DNA sequence analysis.

### Statistical analysis

All data are presented as mean ± standard deviation in at least three experiments in triplicate (*n* ≥ 9). Statistical significance between groups was determined using Student's *t*-test (matched pairs test or unmatched test was used as indicated in figure legends). All tests were performed using the JMP Statistical Discovery™ software by SAS.

## RESULTS

### Recruitment of RA receptor and activation of RNA polymerase II at RA-target promoters

**Figure 1.**
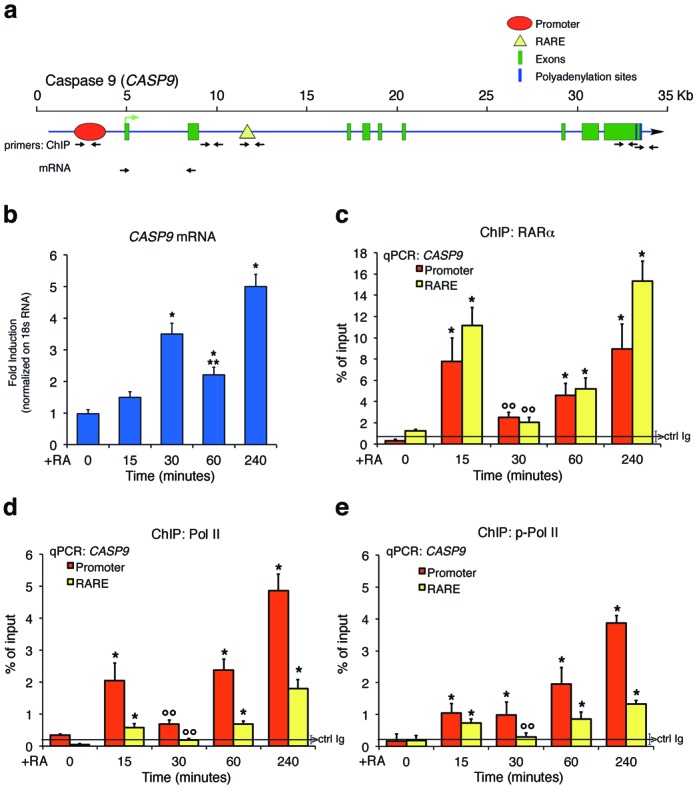
Retinoic acid (RA) induction of *CASP9* mRNA and recruitment of retinoic acid receptor alpha (RARα) and phosphorylated RNA polymerase II to retinoic responsive element (RARE) and promoter of *CASP9* gene. (**a**) Structure of *CASP9* gene. The TSS and the direction of transcription are indicated by a green arrow; the exons, promoter, polyA addition sites and RARE are shown by different colors indicated at the upper right corner. The black arrows indicate the primers used for ChIP and mRNA analysis. (**b**) Total RNA was prepared from MCF-7 hormone-starved or stimulated with 300-nM RA for 15, 30, 60 and 240 min and analyzed by qPCR with specific primers (panel (a)) to *CASP9* mRNA normalized to 18S RNA levels. The statistical analysis derived from at least three experiments in triplicate (*n* ≥9; mean ± SD); *P < 0.01 (matched pairs *t-*test) compared to RA-unstimulated sample, **P < 0.01 (matched pairs *t*-test) comparing 30–60 min of RA exposure. (**c, d, e**) qChip analysis of RA-dependent occupancy of RARα, RNA polymerase II (Pol II) and phosphorylated RNA polymerase II (p-Pol II) at the promoter and RARE. MCF7 cells were stimulated with 300-nM RA for 15, 30, 60 and 240 min. The chromatin was immunoprecipitated with antibodies directed against RARα, Pol II, p-Pol II. Panel (c) shows the recruitment of RARα to the promoter and RARE sequences of *CASP9* gene. Panels (d) and (e) show the recruitment of Pol II (d) and p-Pol II (e) at the promoter and RARE. The black, horizontal line (brackets ± SD) in each plot indicates the percent of input from a control ChIP (Ab: non-immune serum). The statistical analysis derives from at least three experiments in triplicate (*n* ≥ 9; mean ± SD); *P < 0.01 (matched pairs *t-*test) compared to RA-unstimulated sample; °°*P* < 0.01 (matched pairs *t-*test) compared 15–30-min stimulated samples.

The biological activity of RA is mediated by its binding to RA receptors (RARα, RARβ and RARγ), which function as hetero-dimers with RXRs, targeting specific sites (RAREs). We have used breast cancer cells MCF-7, because they express RARs and respond to RA (10). We studied induction by RA of *CASP9* and *CYP2626A1* (Figure 1a and Supplementary Figure S1a) by exposing MCF-7 cells to RA and measuring mRNA levels at different times following stimulation. Figure 1b and Supplementary Figure S1b show that both mRNAs accumulate 30 min after RA exposure. After a transient reduction at 60 min (a slight, but reproducible, decrease in *CYP26A1* mRNA), their levels reached a maximum 4 h after RA exposure. In *CASP9*, a RARE element is localized in intron II, 9.5 kb downstream of the TSS (Figure 1a). *CYP26A1*, instead, contains a RARE contiguous with the promoter (−150 bp from the TSS) and another site at the 3′ end close to the polyA (Supplementary Figure S1a; 11). These sites are essential for RA induction of transcription (4,12,13), which depends on RARα, because under the same conditions, RA is not able to induce *CASP9* and *CYP26A1* mRNAs in MCF10 cells, devoid of RA receptor (Supplementary Figure S1e and f) (14).

To monitor recruitment of the RA receptor to the promoter and RARE elements after RA stimulation, we assessed the timing of association of RA receptor with the chromatin of *CASP9* and *CYP26A1* by ChIP. We included in our analysis very early times after RA induction (min) to detect the earliest chromatin changes induced by the hormone. Figure 1c shows that RARα is rapidly (15 min) recruited to the RARE element and to the upstream promoter of *CASP9*. We noticed that the levels of RARα recruited to the promoter and RARE chromatin were not stable, but oscillated between 15 and 60 min after RA exposure. RARα and RXRα were also recruited to the RARE/promoter of *CYP26A1* (Supplementary Figure S1c and d). RARα did not accumulate to the RARE or promoter of *CASP9* gene in unresponsive MCF10 cells (Supplementary Figure S1f). Recruitment of RARα and RXRα was associated with accumulation of total and serine 5-phosphorylated RNA polymerase II (Pol II and p-Pol II, respectively) at the RARE and promoter regions of *CASP9* and *CYP26A1* (Figure 1d and e, and Supplementary Figure S1d). As expected, Pol II and p-Pol II accumulated preferentially at the promoter relative to the RARE following RA induction. Note that recruitment of total Pol II and p-Pol II to RARE oscillated synchronously with the recruitment of RARα. p-Pol II progressively accumulated at the *CASP9* promoter with the time of RA stimulation (Figure 1d and e, and Supplementary Figure S1d). The levels of p-Pol II at the *CASP9* RARE dipped at 30 min and then increased over the next 240 min, preceding by 30 min the oscillations of the mRNA levels (Figure 1b). The oscillation of RARα recruitment to the *CYP26* RARE promoter–RARE is delayed by 30 min compared to that of *CASP9* and mirrors the smaller and higher peaks of mRNA accumulation at 30 and 240 min following RA exposure (Supplementary Figure S1c and d). mRNA levels of four other genes induced by RA display a similar pattern with a small peak at 30–60 min and a large peak at 240 min after RA (not shown). In *CASP9* normalization of p-Pol II with total Pol II shows that this ratio increases dramatically 30 min after RA at the promoter, while it is stable at the RARE (Figure 1d and e) mirroring the rise and reduction of RNA at 30 and 60-min RA, respectively (Figure 1b and Supplementary Figure S1b). However, we note that at 30 min the change of the ratio is due to a significant depletion of total PolII from the promoter (Figure 1d) which may explain the reduction of mRNA 30 min later (Figure 1b). We believe that the loss of total Pol II at the promoter 30 min after RA does not reflect an increased Pol II phosphorylation, but instead, a change in chromatin structure leading to depletion of Pol II with other proteins bound to the promoter, including RARα (Figure 1c).

### Histone H3K4 and H3K9 methylation marks induced by RA

**Figure 2.**
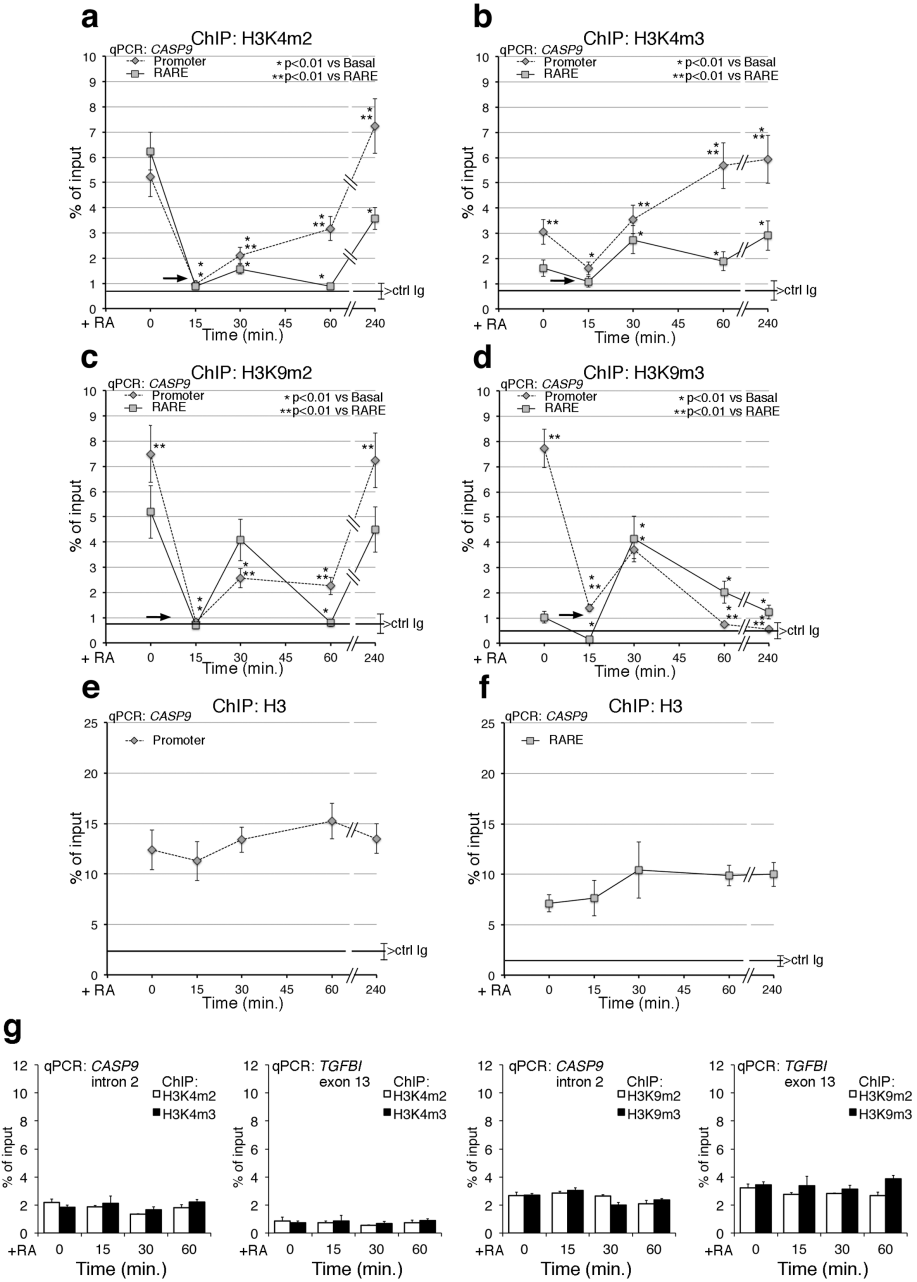
Methylation–demethylation cycles of histone H3K4/K9 induced by RA at *CASP9* promoter–RARE chromatin. MCF7 cells were serum starved and exposed to 300-nM RA at the indicated times (0, 15, 30, 60 and 240 min). qChIP was carried out using specific antibodies recognizing H3K4me3, H3K4me2, H3K9me3 and H3K9me2. The specificity of the antibodies was tested by competition with the specific methylated peptide(s). (**a, b**) H3K4me2 and H3K4me3 occupancy at the *CASP9* promoter and RARE. (**c, d**) H3K9me2 and H3K9me3 occupancy at the *CASP9* promoter and RARE. The black arrows indicate the loss of all methylated H3K4 and H3K9 (except H3K4me2 at the RARE) at 15-min RA**.** (**e, f**) ChIP Histone H3 at the promoter and RARE after RA induction. (**g**) ChIP analysis of *CASP9* II intron and of TGFBI exon 13 (non-RA-induced gene), in cells exposed to RA for 15, 30 and 60 min. For each antibody, the control IgG signal is shown (brackets ± SD). The statistical analysis was derives from at least three experiments in triplicate (*n* ≥ 9; mean ± SD); *P < 0.01 (matched pairs *t-*test): compared to the RA-unstimulated sample; **P < 0.01 (student *t-*test): compared to the same time point at the promoter and RARE.

Methylation of lysine 4 in histone H3 (H3K4) marks transcribed loci, whereas dimethyl-lysine 9 in the same histone (H3K9me2) is associated with transcription silencing (15). To find the histone marks modified by RA exposure, we analyzed the methylation profiles of H3K4 and H3K9 in cells after treatment with RA. ChIP analysis was performed with specific antibodies against methylated H3K4 and H3K9. The regions analyzed were the promoter-TSS, the RARE element and two polyA addition sites located at the 3′ end of the *CASP9*. Two segments of the gene 2 kb distant from the TSS were also included in the analysis. As a result of methylation and demethylation events induced by RA, the levels of methylated K9 or K4, normalized to the input chromatin, were selectively modified shortly after RA exposure. Figure 2 shows that promoter-associated H3K4 me2 and me3 were transiently demethylated 15 min after RA challenge and then progressively remethylated (Figure 2a and b). Rapid demethylation of H3K4 at RARE was also observed, but remethylation was slower than at the promoter. RA induced a transient loss of H3K9me2 and H3K9me3, followed by accumulation of H3K9me2 but not of K3K9me3 (Figure 2c and d). The methylation changes observed were not due to loss of H3 levels at these sites (Figure 2e and f). These cyclical methylation–demethylation events were strikingly synchronous and specific to the RARE and promoter regions of *CASP9*. In contrast, H3 methylation was unaffected by RA at a site 2 kb downstream of RARE in intron II or in a non-RA-induced gene, TGFBI (Figure 2g). A similar methylation–demethylation cycle was observed at the *CYP26A1* promoter–RARE chromatin (Supplementary Figure S2a and b). MCF10 cells, which do not respond to RA (14; Supplementary Figure S1e and f), do not show changes in methylation of H3K4, K9 me2/3 at 15-min RA (Supplementary Figure S2c). We also probed the 3′ end of *CASP9* gene, where two major polyA addition sites are located. H3K4me2 and me3 and H3K9me2 at the polyA1 and polyA2 sites also underwent transient and early demethylation. H3K9me3 was permanently demethylated at the polyA2 site, but was essentially unchanged at the polyA1 site (Supplementary Figure S2d–i). We conclude that the polyA1 and polyA2 sites undergo methylation changes similar to those seen at the promoter and RARE, raising the possibility that these regions are functionally and physically associated in a unique chromatin domain.

**Figure 3.**
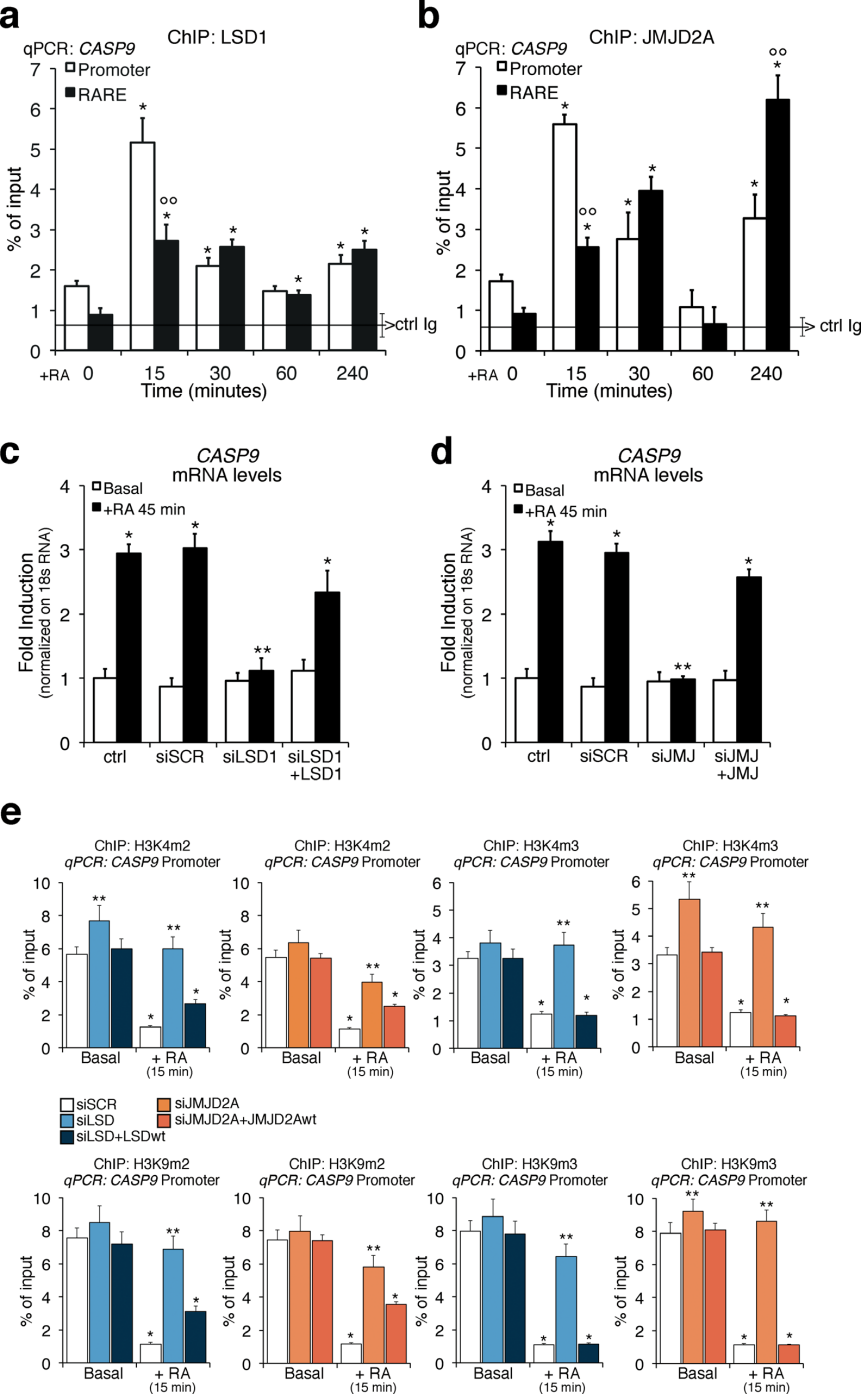
LSD1 and JMJD2A upon RA exposure are recruited to the *CASP9* promoter and RARE sites and are essential for *CASP9* induction by RA and methylation–demethylation cycle. (**a, b**) Recruitment of LSD1 and JMJD2A to the promoter and RARE of *CASP9* gene. MCF7 cells were serum starved and exposed to 300-nM RA at the indicated times (15, 30, 60 and 240 min). qChIP was carried out using specific antibodies recognizing LSD1 and JMJD2A. The panels show the time course of the recruitment of LSD1 (a) and JMJD2A (b) to the RARE and promoter sequences of *CASP9* analyzed by qPCR. The black, horizontal line in each plot indicates the percent of input from a control ChIP (Ab: non-immune serum). (**c, d**) LSD1 and JMJD2A depletion inhibits RA-induced transcription of *CASP9*. MCF7 were transiently transfected with LSD1 siRNA or JMJD2A siRNA. After 48 h, total RNA was prepared from control cells (starved) or RA-induced cells (300-nM RA for 45 min) and analyzed by qPCR with specific primers to *CASP9* mRNA. The statistical analysis derives from at least three experiments in triplicate (*n* ≥ 9; mean ± SD); *P < 0.01 (matched pairs *t-*test) compared to RA-unstimulated sample; **P < 0.01 (student *t-*test): comparison between SCR and specific siRNAs; °°*P* < 0.01 (student *t-*test): comparison between promoter and RARE regions. (**e**) Depletion of LSD1 or JMJ2DA inhibits the methylation changes induced by RA. qChIP was performed on cells transfected with LSD1 siRNA or JMJD2A siRNA and induced with 300-nM RA for 15 min. qChIP was carried out using specific antibodies recognizing H3K4me3, H3K4me2, H3K9me3 and H3K9me2. The statistical analysis derives from at least three experiments in triplicate (*n* ≥ 9; mean ± SD); *P < 0.01 (matched pairs *t-*test) compared to RA-unstimulated sample; **P < 0.01 (student *t-*test): comparison between SCR and specific siRNAs. Transfection efficiency was monitored by FACS (Alexa Fluor or co-transfected pEGFP Vector).

To complete the description of chromatin factors directly or indirectly linked to the modification of histone marks following RA induction of transcription, we measured the recruitment of a major histone methyltransferase (SUV39H1, which methylates H3K9) (16,17) (Supplementary Figure S3a and b) or an alternative modification of H3K9, such as H3K9 acetylated (H3K9Ac) (Supplementary Figure S3c and d), or the recruitment of the co-repressors, NCoR1 and NCoR2 (18,19) (Supplementary Figures S3e and f and S3g and h, respectively) to the RARE–promoter and polyA addition sites of *CASP9* and *CYP26A1* genes. Supplementary Figure S3 shows that the recruitment of these proteins to these sites following RA induction mirrors the demethylation–methylation cycles described above. SUV39H1 accumulates at the promoter, RARE and polyA addition sites with a sharp peak 30 min following RA exposure (Supplementary Figure S3a and b). The SUV39H1 recruitment overlaps with the peak at 30 min of bi- and tri-methylated H3K9 in Figure 2c and d. On the other hand, H3K9Ac oscillates symmetrically with the demethylation wave of H3K9me2 and H3K9me3, suggesting that H3K9 nucleosomes undergo mutually exclusive demethylation–acetylation cycles (Supplementary Figure S3c and d). NCoR1 is transiently recruited at 15-min RA, while NCoR2 is present in the absence of RA and progressively disappears from the RARE chromatin following RA induction (Supplementary Figure S3e–h). Collectively, these data describe a series of intertwined oscillating cyclic events driven by RA and targeted to the RARE–polyA addition sites of *CASP9* and *CYP26A1* genes. The earliest temporal event we are able to detect following RA exposure is the massive demethylation of H3K4 and H3K9 induced by RA at 15 min. This suggests that K4 and K9 demethylation enzymes are also recruited to *CASP9* and *CYP26A1*. H3K9me3 can be demethylated by enzymes of the Jumonji C (JMJC) family (20,21), whereas H3K4me2 is demethylated *in vitro* (22) and H3K9me2 *in vivo* (7,23–25) by LSD1 (KDM1). We decided to monitor the recruitment at the RARE promoter chromatin of *CASP9* and *CYP26A1* of LSD1 and JMJD2A, which demethylate H3K4me2 or H3K9me2 and H3K9me3, respectively. Figure 3a, b and Supplementary Figure S4a show recruitment of both LSD1 and JMJD2A histone demethylases to the RARE elements and promoters of *CASP9* and *CYP26A1* following RA treatment. Notably, the kinetics of recruitment of LSD1 and JMJD2A parallels the kinetics of loss of the H3K4me2 and H3K9me2/3. H3K9me2/3 accumulation at 30-min RA is associated with the recruitment of SUV39H1 (16) to the promoter, RARE and polyA addition sites (Supplementary Figure S3a and b). A new wave of demethylation of H3K9me3 occurs at 60–260-min RA as indicated by reduction of H3K9me3 and accumulation of H3K9me2 (Figure 2c and d). The methylation changes (H3K9me2/3) and the kinetics of LSD1 and JMJD2A recruitment to *CYP26A1* chromatin are very similar to those seen at RARE (Figure 2b and Supplementary Figures S2b, 3a and b and S4a). We believe that this similarity is due to the fact that the promoter and RARE are physically contiguous in *CYP26A1* but are separated in *CASP9*.

To demonstrate that both lysine demethylases were necessary for RA-induced transcription, we knocked down *LSD1* and *JMJD2A* with specific siRNAs and induced the cells with RA (Supplementary Figure S4b and c). Knock down of either demethylase, as shown by retention of di- and tri-methylated H3K4 and H3K9, significantly reduced RA-induced expression of *CASP9* (Figure 3c and d) and *CYP26A1* (Supplementary Figure S4d and e). Re-expression of the wild-type LSD1 or JMJD2A and not a mutant form of these enzymes restored RA-induced *CASP9* RNA levels (Figure 3c and d and data not shown) and reduced the levels of H3K4 and H3K9 both me2 and me3 as in control cells (Figure 3e). These data were unexpected because LSD1 and JMJD2A do not demethylate H3K4me3, although JMJD2A has been shown to bind H3K4me3 *in vitro* (26,27). We suggest that JMJD2A targets to H3K4me3 another enzyme able to demethylate H3K4me3. Also, silencing of either *LSD1* or *JMJD2A* increases H3K4me3 levels, as a result of increased methylation of the me2 forms, which accumulate when LSD1 is not functional.

**Figure 4.**
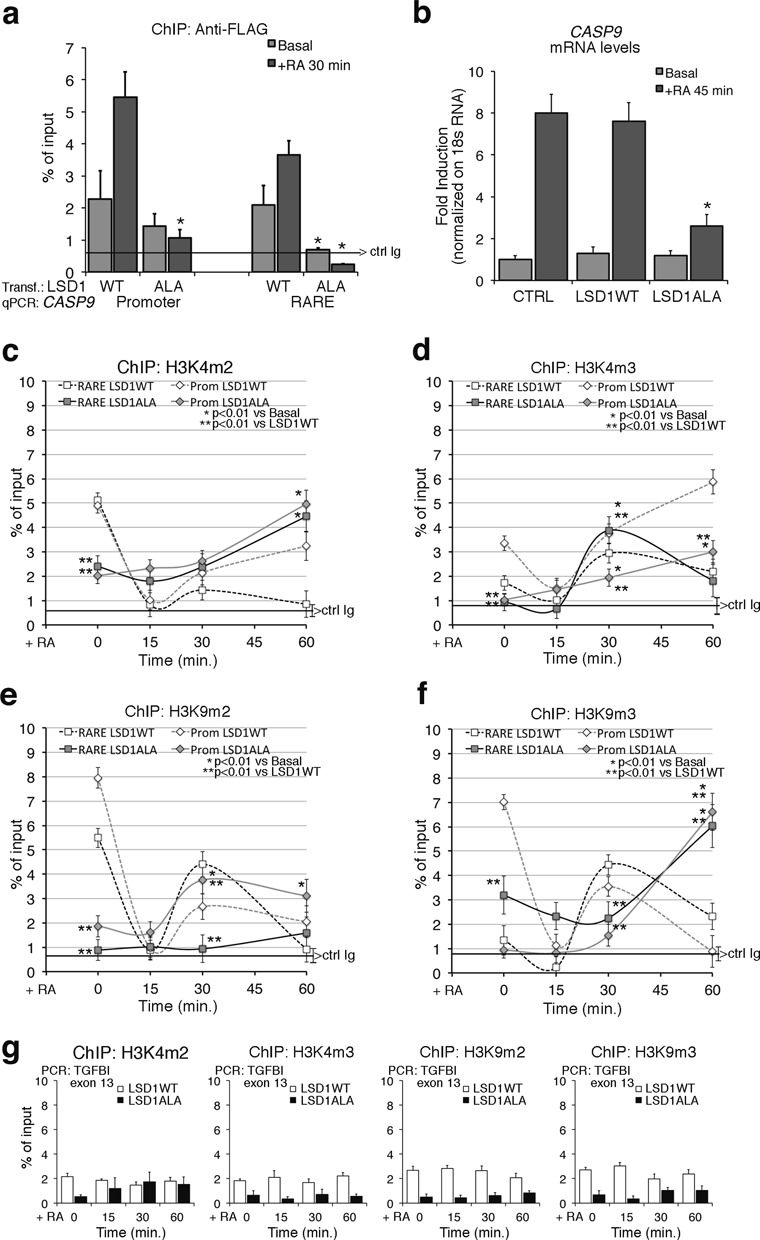
Expression of a dominant negative LSD1 mutant inhibits RA-induced methylation changes and *CASP9* induction by RA. (**a**) Recruitment of wild type (WT) and mutant LSD1 (LSD1ALA) to the *CASP9* RARE–promoter. The LSD1ALA mutant contains a substitution of threonine with alanine in position 110 and has been described elsewhere (17,19). MCF7 were transiently transfected with LSD1 vectors (WT or mutant), starved (Basal) or treated with 300-nM RA for 30 min and were analyzed by qChIP using Anti-FLAG antibodies to recognize the recombinant LSD1. The panel shows the recruitment of the flagged LSD1WT and LSD1ALA to the RARE and to the promoter sequences of *CASP9*. The black, horizontal line indicates the percent of input from a control ChIP (Ab: non-immune serum). (**b**) LSD1ALA inhibits RA-induced *CASP9* transcription. Total RNA was prepared from MCF7 transiently transfected with LSD1WT or LSD1ALA. After 48 h, mRNAs from control cells (starved) or RA-induced cells (300-nM RA for 45 min) were analyzed by qPCR with specific primers to *CASP9* mRNA. (**c, d**) LSD1ALA inhibits RA-induced H3K4 demethylation. H3K4me2 and H3K4me3 occupancy at the promoter and RARE of *CASP9* in LSD1ALA expressing cells as in (b). (**e, f**) LSD1ALA inhibits RA-induced H3K9 demethylation. H3K9me2 and H3K9me3 occupancy at the promoter and RARE of *CASP9* in LSD1ALA expressing cells as in (b). (**g**) ChIP analysis of TGFBI exon 13 (non-RA-induced gene), in transfected cells exposed to RA for 15, 30 and 60 min. For each antibody, the control IgG signal is shown (brackets ± SD). The statistical analysis derives from at least three experiments in triplicate (*n* ≥ 9; mean ± SD); *P < 0.01 (matched pairs *t*-test): compared to the RA-unstimulated sample; **P < 0.01 (student *t-*test): comparison between control and the LSD1ALA expressing cells at the same time.

To explore further the relationship between H3K9 and H3K4 methylation and LSD1, we overexpressed an N-terminal dominant-negative mutant (T110A) of LSD1 (LSD1ALA). This mutant is still enzymatically active, but is unable to target transcription factors (7,8). The LSD1ALA mutant protein was defective in binding to the promoter or RARE elements of *CASP9* or *CYP26A1* following RA induction (Figure 4a and Supplementary Figure S5a, respectively) and inhibited activation of *CASP9* or *CYP26A1* transcription upon RA exposure (Figure 4b and Supplementary Figure S5b). The methylation levels both H3K4 and H3K9 me2 were already low in the absence of RA (basal in Figure 4c–f) because LSD1ALA was constitutively active and not inducible by RA (Supplementary Figure S5c and d). LSD1ALA was constitutively recruited to chromatin because not only it inhibited RA induction of *CASP9* or *CYP26A1* expression but also lowered the levels of methylated H3K4me2 or H3K9me3 at promoters of non-RA-induced genes (such as *TGFBI*; Figure 4g), which methylation status does not change upon RA induction. However, the methylation–demethylation cycle at the RARE, promoter and polyA addition sites (Figure 4 and Supplementary Figure S56) was abolished both in LSD1 and JMJD2A-depleted cells (Figure 3e) and in LSD1ALA expressing cells (Figure 4c–f) and was not dependent upon the basal methylation levels of H3K4 and H3K9 (me2/3), which were high in the former and low in the latter cells, respectively (Figures 3e and 4c–f).

Collectively, these data indicate that RA induces the recruitment of both LSD1 and JMJD2A to the chromatin of *CASP9* and *CYP26A1* and demethylation of H3K4me2 and H3K9 me2/me3 at the RARE, promoter and polyA2 sites. These localized demethylation events by both demethylases are essential for the induction of transcription of *CASP9* and *CYP26A1* by RA (Figure 4b and Supplementary Figure S5b).

### Recruitment of base or nucleotide excision repair enzymes to the RARE/promoter chromatin following RA induction

**Figure 5.**
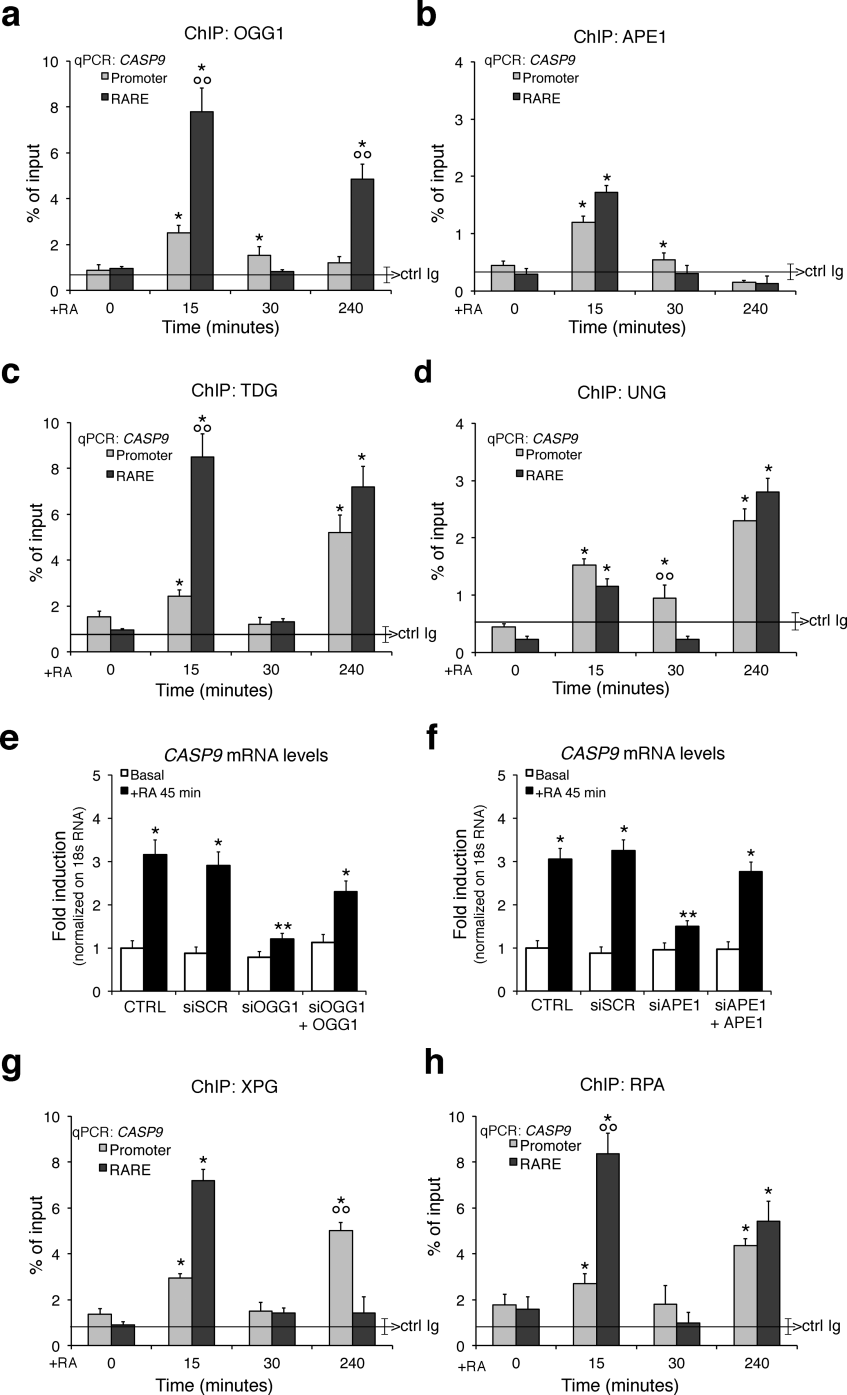
Recruitment of BER and NER enzymes to *CASP9* chromatin following RA induction. MCF7 cells, starved or treated with RA for 15, 30 and 240 min, were analyzed by qChIP using specific antibodies recognizing the 8-oxoguanine-DNA glycosylase-1 (OGG1), AP endonuclease (APE1), Thymine-DNA glycosylase (TDG), Uracil-DNA glycosylase (UNG), XPG and RPA. (**a, b**) The recruitment of OGG1 and APE1 to the *CASP9* promoter, and RARE sequences. (**c, d**) The recruitment of TDG and UNG to the same regions of *CASP9*. The black, horizontal line in each plot indicates the percent of input from a control ChIP (Ab: non-immune serum). (**e, f**) BER knockdown impairs the expression of RA-induced *CASP9*. Serum-deprived MCF7 cells were treated for 45 min with 300-nM RA and specific siRNA targeting OGG1 or APE1; *CASP9* expression levels were quantified by qPCR. To assess the transfection efficiency cells were co-transfected with pEGFP Vector (Clontech) and analyzed by FACS. (**g, h**) The recruitment of XPG and RPA to the *CASP9* promoter and RARE. The statistical analysis derives from at least three experiments in triplicate (*n* ≥ 9; mean ± SD); *P < 0.01 (matched pairs *t*-test) compared to RA-unstimulated sample; **P < 0.01 (student *t-*test): comparison between SCR and specific siRNA; °°*P* < 0.01 (student *t-*test): comparison between the two amplicons.

It was recently reported that nucleotide excision repair (NER) enzymes are recruited to promoter and RARE elements. NER is essential for the formation of discrete chromatin loops and induction of transcription by RA (28). Similarly, transcription-induced recruitment of base excision repair (BER) enzymes (such as 8-oxoguanine-glycosylase, OGG1) to *MYC*-Ebox DNA or to estrogen responsive elements has been described (25,29). Activation of a Fe^++^ dioxygenase (JMJD2A) and a FAD oxidase (LSD1) at the same chromatin sites (ERE or Ebox-promoters) triggers local oxidation. Oxidized guanine (8-oxo-dG) is recognized by OGG1 (8,25). That oxidation of guanine also occurs after RA induction of transcription is shown in Supplementary Figure S7a. We observed a rapid (15 min) nuclear accumulation of 8-oxo-dG in discrete foci in MCF7 cells exposed to RA. As predicted, production of 8-oxo-dG foci was inhibited by LSD1 knockdown (Supplementary Figure S7b). ChIP analysis showed that OGG1 was recruited to the promoter and RARE elements of *CASP9* (Figure 5a) 15 min following RA treatment. At 30 min, occupancy at these sites by OGG1 markedly decreased. At 240 min, OGG1 was detected at the RARE element but not at the promoter. Similar oscillation of OGG1 binding to the RARE/promoter of *CYP26A1* was also seen (Supplementary Figure S8a). RA induces the binding of OGG1 to the ERE and promoter sites with the same timing of nuclear 8-oxo-dG accumulation (Supplementary Figure S7) indicating that 8-oxo-dG accumulates at specific segments of RARE, promoter and polyA addition sites. In fact, OGG1 recruitment was not seen in non RA-induced genes or in segments distant (1–2 kb) from the TSSs of *CASP9* or *CYP26A1* (Supplementary Figure S8g).

Complexes enucleated by OGG1 seem important not only for the repair of oxidized lesions but also for assembly of transcription initiation complexes at estrogen- or Myc-dependent promoters (8,25,30,31). To dissect the components of the OGG1 complex that could link repair and RA-induced transcription, we probed for other BER enzymes that associate with a RARE element or its cognate promoter after RA induction. Specifically, we monitored the recruitment of: (i) the **AP**urinic site **E**ndonuclease1, APE1, which recognizes the apurinic site generated by OGG1 and cleaves the phosphodiester backbone, immediately 5′ to the site; (ii) thymine DNA glycosylase (TDG), which is required for BER of deaminated methylcytosine, a frequent product of base oxidation; and (iii) uracil glycosylase (UNG), which removes uracil or oxidized cytosine. Figure 5b, c and d (Supplementary Figure S8a for *CYP26A1*) show that all these enzymes are recruited to the promoter and to RARE chromatin 15 min following RA induction similar to the recruitment of RARα and Pol II (compare Figure 5 with Figure 1). We knocked down two of these enzymes (OGG1 and APE1; Supplementary Figure S8b and c) and asked if this impacted on RA-induced transcription. Figure 5e and f and Supplementary Figure S8d–f summarize the results of these experiments. The activity of OGG1 and APE1 was essential for RA-induced transcription, since the wild type, but not a OGG1 dominant negative or a catalytically defective mutant APE1, reactivate *CASP9* transcription induced by RA in depleted cells (Figure 5e and f; Supplementary Figure S8d and e and data not shown).

Our results clearly indicate that depletion of these BER enzymes significantly reduced RA-induced transcription. We also probed for an NER enzyme (XPG) and for RPA, a single strand binding protein (Replication Protein A) involved in NER, on RARE by ChIP. XPG and RPA selectively accumulated at RARE chromatin following 15 min of RA stimulation (Figure 5g and h and Supplementary Figure S8a). It has been reported recruitment of NER enzymes to the RARE or other inducible promoters 3–4 h after hormonal induction, a period corresponding to maximal accumulation of specific mRNA levels (28). As expected, depletion of XPG and RPA reduced RA-induced transcription (not shown; 28). Our results show that BER and NER accumulate early at the RARE and promoter, shortly before we could detect mRNA accumulation (Figure 1b and Supplementary Figure S1b). The modifications we describe mark the first transcription cycle of *CASP9* and *CYP26A1* induced by RA.

### Formation of dynamic chromatin loops governing the selection of 5′ and 3′ borders of RA-induced transcription units

The data shown above indicate that the *CASP9* and *CYP26A1* promoter, RARE and polyA addition sites undergo similar changes in histone H3K4 and H3K9 methylation and accumulate BER and NER enzymes after RA treatment. This coordination is consistent with the idea that these regions are physically associated after RA induction. Note that the *CASP9* RARE and 5′ start site are 9.5 kb apart and the polyA site is 22 kb to the 3′ end of RARE (Figure 1a). Recall that the methylation status H3K4 and H3K9 was not modified at chromatin neighboring these sites (2 kb at the 5′ and 3′ ends) and BER–NER enzymes were not recruited to these sites in RA-treated cells (Figure 2e and Supplementary Figure S8g).

**Figure 6.**
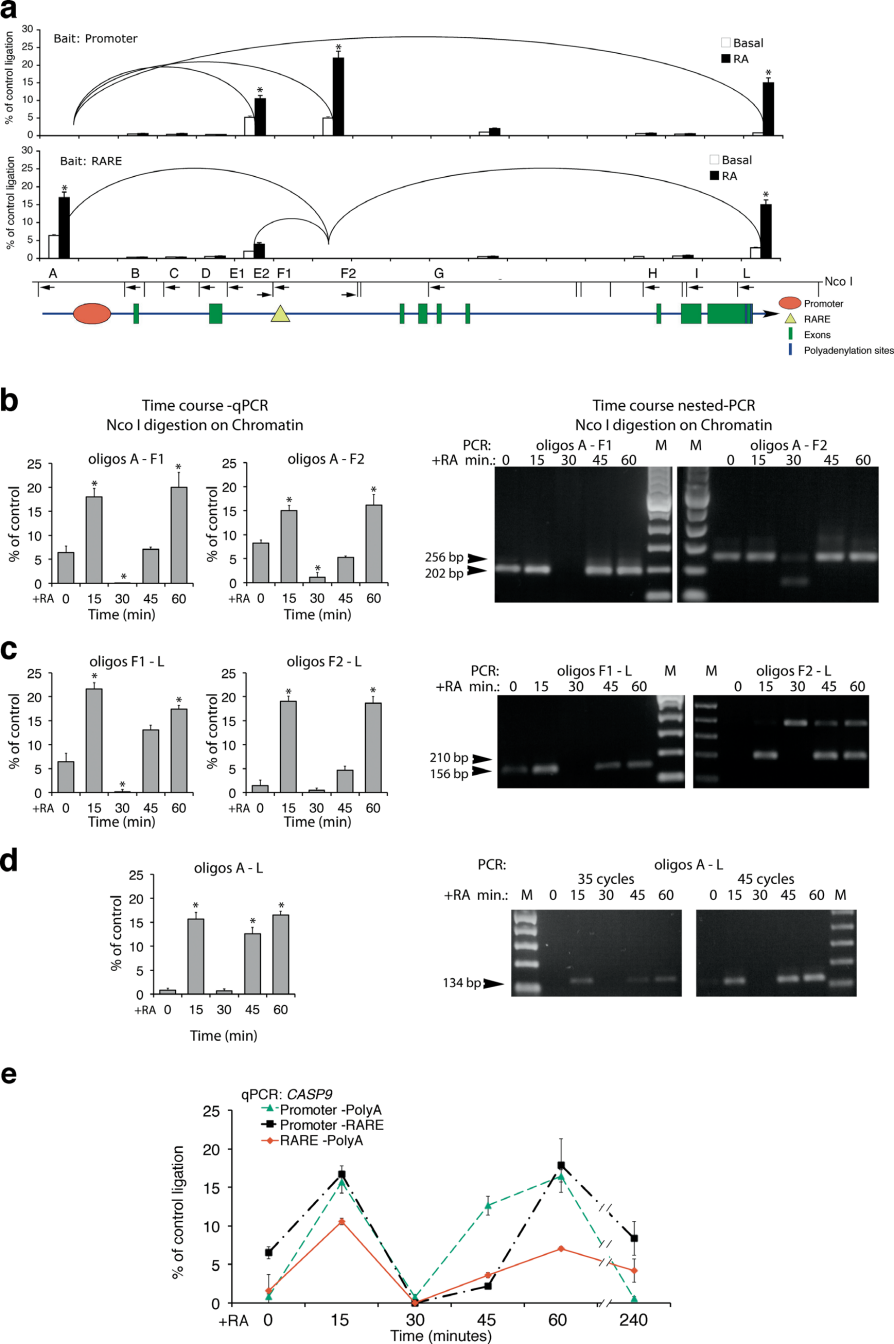
Formation of dynamic chromatin loops during early RA-induced transcription. 3C analysis of *CASP9* chromatin in MCF7 cells exposed to 300 nM of RA for various periods of time. (**a**) The histograms show the frequency of ligation of the *CASP9* NcoI fragments amplified with primers indicated below the NcoI restriction map. All the combinations of primers indicated were performed on ligated chromatin; the histogram shows qPCR amplifications above 1%, relative to the control. Each loop was detected with different primers pairs and the two histograms show the analysis by using several probes and ‘baits’ centered at the TSS, RARE and 3′ end of the *CASP9*. Differences between recombinant, Basal and RA-treated cells derived-chromatin were tested for statistical significance using Student's *t-*test: *P < 0.01 as compared to untreated control. (**b, c, d**) Time course of chromatin looping following RA induction. 3C analysis was carried out as described in the Materials and Methods section and the loops shown in panels (b, c, d) were quantified by qPCR (left panels) and verified by gel electrophoresis (right panels) and DNA sequencing (data not shown). The results shown derive from at least three experiments in triplicate (*n* ≥ 9; mean ± SD). *P < 0.01 as compared to untreated samples. (**e**) The panel shows the time course of loop formation. Data were collected from real-time qPCR and from semi-quantitative, nested PCR, experiments. The extra band seen in panel (c) is a skipped restriction site that adds 187 bp to the expected fragment (210 bp). This band is specific because the same loop (RARE-polyA) analyzed with another primer (F1) does not produce extra bands. Also, following the treatment with RA, this longer fragment accumulates while the shorter one (cleaved by the enzyme in the chromatin) almost disappears. This indicates that this site is protected *in vivo* and is not cleaved efficiently by NcoI restriction enzyme.

These data suggest that RA induces early changes of specific chromatin domains that bring the TSS, the RARE and the polyA addition sites into close proximity. To find the relevant chromatin domains assembled in response to RA, we systematically analyzed the structure of *CASP9* (Figure 6a) and *CYP26A1* (Supplementary Figure S9a) chromatin by the 3C technique (see the Materials and Methods section). Briefly, fixed chromatin DNA was cleaved with a restriction enzyme (NcoI) and ligated after dilution. Real-time qPCR was then used to detect the ligated DNA segments. Figure 6a shows the summary of such analysis by using several probes and ‘baits’ centered on the TSS, RARE and 3′ end of *CASP9* (see the legend of Figure 6 for the detailed band analysis).

RA enhanced formation of two loops connecting the 5′ and 3′ ends of the gene with the RARE element. Extensive quantitative analysis of these loops revealed: (i) a 5′ end loop connecting the RARE to the promoter (A-F1, A-F2) was induced by RA (Figure 6b); (ii) a loop (F1-L, F2-L) connecting the RARE region to the 3′ end of *CASP9*, where two different polyA addition sites generate two mRNA ends (AB209147; AK303743). Assembly of this loop was almost entirely dependent on RA treatment (Figure 6c); (iii) a loop connecting the 5′ and the 3′ ends of the gene, bridging the above-mentioned loops (A-L) (Figure 6d). We wish to note that transcription of *CASP9* is promoted by many different stimuli. Thus, the loops formed in the absence of RA may nevertheless reflect basal transcription.

Strikingly, formation of all of these loops is cyclical. They first appear 15 min following RA exposure, disappear at 30 min and reform by 60 min. This oscillation resembles that displayed in previous figures showing *CASP9* mRNA synthesis, promoter and RARE occupancy by protein factors and histone modifications (Figures 1–5). The *CYP26A1* gene also formed chromatin loops upon RA treatment. Since RARE and promoter are contiguous in *CYP26A1* we detected essentially one major loop connecting the 5′ (promoter-RARE) with the 3′ end of the gene (polyA addition sites). This loop peaked 15 min after RA and slowly disappeared (Supplementary Figure S9b and c) similar to the early loop induced by RA on *CASP9* chromatin (Figure 6e). The physical association of the 5′ and 3′ ends of *CASP9* and *CYP26A1* genes induced by RA implies that the same proteins are present at the chromatin of the promoter, RARE and 3′ end sites. The physical contiguity (600 bp) of the two polyA addition sites (1 and 2) does not discriminate which polyA1 or 2 is included in the RA-induced loop of *CASP9* gene.

To find a 3′-end-specific RA-dependent marker of *CASP9*, we investigated the localization on *CASP9* chromatin of Ssu72, a protein which marks the 3′ end of genes and interacts with the general transcription initiation factor, TFIIB (32). Supplementary Figure S10a, b and c shows that Ssu72 binds the promoter and RARE with the same kinetics seen with RARα and Pol II following RA exposure, i.e. a peak at 15 min corresponding to the early RA-induced loop (Figure 6e). At 15 min after RA, Ssu72 disappeared from the polyA2 and concentrated at the promoter and RARE (Supplementary Figure S10a and b). Apparently, Ssu72 was present at the polyA2 site of *CASP9* gene in the absence of RA induction, except at 15′ min after RA, when the receptor and the promoter were recruiting Pol II and RARα (Supplementary Figure S10a–c).

How relevant are these loops to RA-induced transcription and how they are linked to the demethylation cycles induced by RA? To address this question we measured the loops involving RARE in cells expressing the LSD1ALA mutant. Expression of LSD1ALA inhibited RA-induced demethylation (Figure 4c–f and Supplementary Figure S6) and RA-induced transcription (Figure 4b). Supplementary Figure S10d shows that the formation of the 15-min loops connecting RARE to the polyA1/2 site or to the promoter upon RA exposure was inhibited: some loops were delayed (RARE-polyA1/2) and some others were completely eliminated (RARE–promoter).

We conclude that the demethylation cycles triggered by RA govern the ordered formation of the loops essential for RA-induced transcription.

## DISCUSSION

The data reported here show that the methylation changes of K4 and K9 of histone H3 are linked to the recruitment of repair enzymes and, most importantly, to the formation of chromatin-DNA loops that juxtapose the 5′ end TSS, the enhancer (RARE) and the 3′ end of the transcribed gene (Figure 6 and Supplementary Figures S9 and S10). Histone methylation–demethylation cycles (33) and the formation of loops connecting the 5′ gene ends, 3′ ends and enhancers have been described extensively in many genes induced by nuclear receptors (28,33). However, these aspects of chromatin movement, although required for transcription induction by nuclear hormones, have not been mechanistically and temporally linked. Likewise, the association of methylation–demethylation cycles with NER enzyme recruitment to RA-induced promoter(s) and the formation of loops upon exposure to RA has not been described, although depletion of these enzymes seriously compromises transcription and chromatin looping induced by RA (28). However, notwithstanding the plethora of data, the mechanism used by RA or other inducers to trigger the recruitment of NER enzymes and formation of chromatin loops is still not known. Our experiments show that demethylation–methylation cycles of H3K4 and K9 at the RARE, promoter (Figure 2) and 3′ gene end (Supplementary Figure S2d–g) are the initiating and earliest events induced by the recruitment of the RA–RARα complex to the RARE. The timing and the kinetics of demethylation of H3K4 and K9 are similar at the promoter, RARE (Figure 6d) and the 3′ end of the gene, suggesting that these sites, although not contiguous in the DNA, are included in the same complex, driven by active RA–RARα. Inhibition of demethylation of H3K4 and H3K9 by depleting the demethylating enzymes (LSD1 or JMJD2A) or by expressing a dominant negative LSD1 variant (7,8) inhibits RA-induced transcription (Figure 3c and d and Supplementary Figure S4d and e), nuclear dG oxidation (Supplementary Figure S7b), recruitment of BER (25) and NER enzymes and formation of loops induced by RA (Supplementary Figure S10d). Formation of the chromatin loops with discrete 5′ and 3′ borders is facilitated by local DNA oxidation following demethylation by LSD1, which presumably releases supercoiling and rigidity of the helix and targets BER and NER enzymes to oxidized bases (34; Antonio Pezone & Antonio Porcellini, manuscript in preparation). In all cases, BER and NER enzymes are essential to localize and repair DNA nicks and altered bases produced by dG and 5mdC oxidation (25,31,35). We wish to stress that inhibition of H3K4-K9 methylation–demethylation cycles or depletion of BER or NER enzymes or overexpression of LSD1ALA does not influence the expression or modify the chromatin of RA-independent genes (Figures 2g and 4g and Supplementary Figures S4f and g and S8f). Locally, LSD1-triggered demethylation produces hydrogen peroxide, which oxidizes dG (Supplementary Figure S7) and both H3K9me2 and H3K4me2, as LSD1 substrates (7,8,25) may favor dG oxidation. We do not know if H3K9m2/3 coexist with H3K4me2/3 nucleosomes, but our data show a rapid demethylation 15 min after RA, involving both H3K4 and H3K9 N-terminals at the RARE–promoter and polyA addition sites (Figure 2 and Supplementary Figure S2d–g). At these sites, 4 h after RA, the content of H3K9me3 and H3K4me3 is low and high, respectively, as expected in transcriptionally active genes (36). The presence of H3K4me2/3 and H3K9me2/3 at time 0 at the promoter or RARE may indicate that these sites are poised between repression and activation depending on the strength of the specific stimulus. Also, RARE nucleosomes containing H3K9me2 may express less CASP9 and this may contribute to the setting of discrete expression states in cell populations.

Further complicating comparative analysis of the data published thus far are the different temporal frames used in various studies to describe the molecular events induced by transcriptional activators. These studies have been carried out at 1 h (37) to several hours or days (28) after hormonal induction. At these times, synchronization is lost. Each cell is starting and restarting the transcription cycle and only the mature RNA accumulates exponentially and can be easily detected even in asynchronous transcribing cell populations. The H3 (K4-K9) methylation code, hours and days after the initial RA induction, is not informative since H3K4me2/3 and H3K9me2/3 content is high and low both in control and chronically stimulated cells, respectively (33).

An important feature of RA-induced transcription reported here is the timing and synchronous oscillation with a period of ca. 30 min of chromatin bound RARα, Pol II, H3K4-H3K9 demethylation, H3K9Ac and looping involving the RARE and the 5′ and 3′ of *CASP9* and *CYP26A1* genes (Figure 6). RA-induced mRNA levels of *CASP9*, *CYP26A1* and five other genes (A. Pezone, unpublished observations) oscillate with a period of 60 min from the initial induction with RA. With the time (2–4 h) this periodic oscillation is lost (Figure 6). We do not know if this loss of synchrony with the time is due to our inability to track these chromatin changes in non-synchronized cell populations or whether the oscillation we observe is limited to the first transcription cycle. An early and similar cycle of transcription induced by estrogens has been reported to be unproductive in terms of RNA accumulation. This cycle is suggested to prepare the promoter for subsequent transcription followed by two different transcriptionally productive cycles (38–40). However, our data suggest that (i) the first cycle (unproductive in 38–40) is indeed the cycle (15–30 min) that sets and defines the physical borders of the transcription unit induced by the hormone (in our case RA) and (ii) the oscillations in RARα recruitment (Figure 1) and chromatin looping (Figure 6) are driven by methylation–demethylation cycles of histone H3K4 and H3K9, caused by alternate recruitment of demethylating (LSD1-JMJD2A in Figure 3a and b and Supplementary Figure S4a) and methylating (Supplementary Figure S3a and b) enzymes. We believe that these oscillations are important for BER and NER enzymes to repair oxidized DNA (Figure 5 and Supplementary Figure S8a) to restart the cycle.

We wish to note that although the structural organization of the RARE is different comparing *CASP9* (RARE 4 kb downstream the promoter) with *CYP26A1* (RARE contiguous with the promoter) genes, the timing of methylation–demethylation, the oscillation of RARα and LSD1-JMJD2A recruitment to the RARE promoter appear very similar

Why should the definition of the physical borders of a transcription unit be important? Each gene in eukaryotes is indeed the target of many stimuli, which can independently induce transcription. The protein Ssu72 has been identified in yeast as an element required for marking the 3′ end of the chromatin loops and transcription directionality (41,42). The human protein Ssu72, although has not been shown to be involved in DNA-chromatin loops, may have a similar function in human cells as suggested by the crystal structure of the complex of RNA polymerase C terminal domain, a scaffold protein symplekin, and human Ssu72 (43).

**Figure 7.**
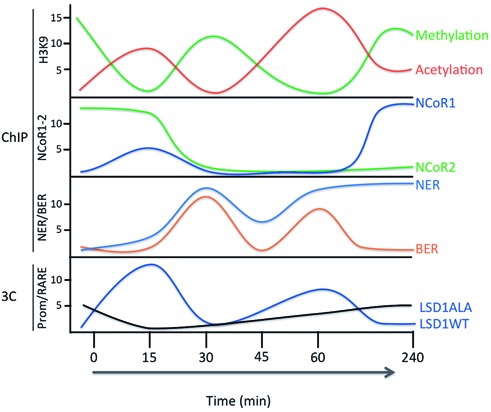
RA-induced cycles of histone H3K9 methylation, acetylation, recruitment of NCoR1–2, BER, NER and RARE–promoter chromatin loop. Time course of the major modifications of histone H3K9 at the RARE of *CASP9* and *CYP26A1* genes. The original data are in Figures 2, 5 and 6 and Supplementary Figures S2, S3, S8, S9 and S10. On the ordinate are indicated: i. fold-induction (arbitrary units); ii. the type of assay (ChIP or 3C). The time of RA induction is shown on the abscissa. The bottom panel shows the frequency of loop formation between RARE–promoter of *CASP9* gene in cells expressing wild type or LSD1ALA (Supplementary Figure S10d).

The accumulation of Ssu72 in the various sites of *CASP9* gene, before and after RA induction, may indicate the changes of chromatin engaged in RA-induced and RA-independent transcription. This protein is present at 3′ end of *CASP9* 3′ end before RA induction, disappears from this site at 15 min and reappears 30 min after RA treatment (Supplementary Figure S10a–c). Fifteen minutes after RA stimulation, Ssu72 moves from the 3′ end to the *CASP9* TSS and RARE (Supplementary Figure S10a–c), where its concentration oscillates synchronously with methylation–demethylation cycles and recruitment of BER and NER enzymes (Figures 2 and 5 and Supplementary Figure S10). These data indicate that Ssu72 specifies the 3′ end of both RA-dependent and -independent transcription units (Supplementary Figure S10c), which are also marked by end- and time-selective-specific chromatin loops. Importantly, after RA induction, RA-independent transcription is shut off to allow the formation of RA-dependent loops. This is shown by the shift of Ssu72 from the polyA2 addition site (in the absence of RA) to the RARE (15 min after RA) (Supplementary Figure S10a–c) and by the time synchrony of the loops induced by RA (Figure 6e). LSD1 is essential for this event, because the synchrony of loop formation is lost in cells expressing the LSD1ALA mutant: some loops disappear (promoter-RARE) or are delayed (promoter-polyA or RARE-polyA) and resemble loops formed in the absence of RA (Supplementary Figure S10d). It is worth noting that LSD1 has been recently shown to control the rhythmicity and the circadian clock and that a mutant in the residue adjacent (aa 111) to that of LSD1ALA (aa 110) is unable to reset clock oscillations (44). We suggest that RA-induced synchronous demethylation–methylation cycles inhibit ongoing local transcription and trigger recruitment of BER and NER enzymes to the chromatin of *CASP9* promoter, RARE and 3′ end. The simultaneous DNA and histone changes bring in close proximity the 5′, enhancer and 3′ ends of *CASP9* and induce the formation of loops (Figure 6 and Supplementary Figure S10). Figure 7 shows a summary of temporal changes induced by RA on the target chromatin, including histone H3K9 acetylation, methylation, demethylation, recruitment of co-repressors NCoR1-2, of BER and NER enzymes and the formation of the loop connecting RARE and promoter in *CASP9* gene. We propose that these cycles are intertwined and triggered by histone methylation–demethylation cycles.

We believe that RA-induced synchronization of DNA chromatin loops is required for calibration and rapid reinduction of transcription rather than for high levels of transcription. The massive accumulation of RA-specific mRNAs, in fact, occurs hours after the initial RA exposure (45). This efficient reinduction of transcription may represent a ‘transcription memory’, which has been noted before and requires gene looping and Ssu72 in yeast (46,47).

The periodic variation of recruited receptor at the enhancer represents a simple mechanism to titrate and calibrate the concentration of RA molecules present in the environment. Any drop in the inducer levels reduces the concentration of active receptor at the enhancer leading to dissolution of the chromatin DNA loop enucleated initially by the active receptor (RARE–promoter). Conversely, a rise in levels of the inducer rapidly reactivates transcription by stabilizing the loops (transcription memory) (see the Supplementary Movie).

## SUPPLEMENTARY DATA

Supplementary Data are available at NAR Online.

SUPPLEMENTARY DATA
