# Supplementary material for: Mechanism of retinoic acid-induced transcription: histone code, DNA oxidation and formation of chromatin loops
Source: Nucleic Acids Res. 2014 Sep 12;42(17):11040–55. doi: 10.1093/nar/gku823 (PMC4176188; doi:10.1093/nar/gku823)
Supplement: SUPPLEMENTARY DATA [file supp_42_17_11040__index.html]

Mechanism of retinoic acid-induced transcription: histone code, DNA oxidation and formation of chromatin loops — Mechanism of retinoic acid-induced transcription: histone code, DNA oxidation and formation of chromatin loops — SUPPLEMENTARY DATA 

# Mechanism of retinoic acid-induced transcription: histone code, DNA oxidation and formation of chromatin loops

## SUPPLEMENTARY DATA

**Files in this Data Supplement:**

- SUPPLEMENTARY DATA
- SUPPLEMENTARY DATA
