# Supplementary material for: Mechanism of retinoic acid-induced transcription: histone code, DNA oxidation and formation of chromatin loops
Source: Nucleic Acids Res. 2014 Sep 12;42(17):11040–55. doi: 10.1093/nar/gku823 (PMC4176188; doi:10.1093/nar/gku823)
Supplement: SUPPLEMENTARY DATA [file supp_gku823_nar-01465-x-2014-File009.pdf]

Supplemental Information for

Mechanism of Retinoic acid-induced transcription: histone code, DNA oxidation and formation of chromatin loops

Candida Zuchegna[1], Fabiana Aceto[2], Alessandra Bertoni[3], Antonella Romano[1], Bruno Perillo[4], Paolo Laccetti[1], Max E. Gottesman[5], Enrico V. Avvedimento[3], Antonio Porcellini[1]

1. Dipartimento di Biologia, Università Federico II, 80126 Napoli, Italy
2. Dipartimento di Medicina e di Scienze della Salute, Università del Molise, 86100 Campobasso, Italy
3. Dipartimento di Medicina Molecolare e Biotecnologie mediche, Istituto di Endocrinologia ed Oncologia Sperimentale del C.N.R., Università Federico II, 80131 Napoli, Italy
4. Istituto di Scienze dell'Alimentazione; C.N.R., 83100 Avellino, Italy
5. Institute of Cancer Research, Columbia University Medical Center, New York, New York, USA

Supplement Index:

Supplementary Methods	page 02
Summary of Supplementary Tables and Figures	page 03
Supplementary Table S1	page 04
Supplementary Figures S1 to S10	pages 5-14

Supplemental Methods

PCR and qPCR protocols.

Real Time-qPCRs were performed using FastStart Universal SYBR Green Master (Rox) (Roche Applied Science) with cycle conditions as follows:
CASP9 Promoter: 95 °C 10 min; 5x (95 °C 45 sec, 68 °C 30 sec, 72 °C 30 sec); 40x (95 °C 45 sec, 65 °C 30 sec, 72 °C 30 sec); 72 °C 10 min.
CASP9 Other regions: 95 °C 10 min; 5x (95 °C 45 sec, 59 °C 30 sec, 72 °C 30 sec); 40x (95 °C 45 sec, 56 °C 30 sec, 72 °C 30 sec); 72 °C 10 min.
CYP26A1 RARE/Promoter region: 95 °C 10 min; 45x (95 °C 45 sec, 56 °C 30 sec, 72 °C 35 sec); 72 °C 10 min.

PCR were performed using FastStart Taq DNA Polymerase (Roche Applied Science) with cycle conditions as follows:

CASP9 oligo A-F1, F1-L, F2-L, A-L: 95 °C 5 min; 5x (95 °C 45 sec, 55 °C 30 sec, 72 °C 30 sec); 30x (95 °C 45 sec, 52 °C 30 sec, 72 °C 30 sec); 72 °C 10 min.

CASP9 oligo F2-L: 95 °C 5 min; 5x (95 °C 45 sec, 54 °C 30 sec, 72 °C 35 sec); 30x (95 °C 45 sec, 51 °C 30 sec, 72 °C 35 sec); 72 °C 10 min.

siRNAs: LSD1A, GS23028 (Qiagen Inc., USA); JMJD2A, SR306452C; OGG1, SR303282; APE1, SR300230; OriGene Technologies, Inc., USA

Antibodies used for the experiments. RARα sc-551 (Santa Cruz Biotechnology) and sb41934 (Abcam); PolII 05-623 (Upstate) ; P-Pol II 04-1572 (Upstate); H3K4me2 ab32356 (Abcam); H3K4me3 ab1012 (Abcam); H3K9me2 ab1220 (Abcam); H3K9me3 ab8898 (Abcam); H3K9Ac ab4441 (Abcam); Total H3 ab1791 (Abcam); NCoR1 ab24552 (Abcam); NCoR2 ab24551 (Abcam); KMT1A/SUV39H1 ab12405 (Abcam); LSD1 sc-271720 and sc-67272 (Santa Cruz Biotechnology); JMJD2A sc-135065 (Santa Cruz Biotechnology); Anti-FLAG F7425 (Sigma-Aldrich); OGG1 sc-33181 (Santa Cruz Biotechnology); TDG sc-22845 (Santa Cruz Biotechnology); UNG sc-28719 (Santa Cruz Biotechnology); APE1 ab-194 (Abcam); RPA sc-14691 (Santa Cruz Biotechnology); XPG sc-73274 (Santa Cruz Biotechnology); SSU72 sc-69613 (Santa Cruz Biotechnology); RXRα sc-553 (Santa Cruz Biotechnology); Normal rabbit IgG sc-2027 (Santa Cruz Biotechnology); Normal mouse IgG sc-2025 (Santa Cruz Biotechnology).

LSD1 Activity/Inhibition Assay. MCF-7 cells were serum starved for 2 days and treated with RA for the indicated times. Untreated or treated MCF-7 cells were washed three times with ice-cold PBS pH 7.4, scraped and lysed in buffer 1 containing 20 mM Tris pH 7.5, 10 mM KCl, 2 mM EDTA, 2 mM $MgCl_2$. After 10 seconds at 12,000 xg at 4 °C, the pellets were resuspended in a buffer 2 containing 20 mM Tris pH 7.5, 400 mM NaCl, 2 mM EDTA, 1 mM $MgCl_2$. After 10 minutes at 12,000 x g at 4 °C, the supernatants were assayed for LSD1 activity. The activity was measured by the EpiQuik™ Histone Demethylase (H3K4 Specific) Activity/Inhibition Assay Kit, according to the manufacturer's instructions (Epigentek, P - 3017; USA).

Buffers Formulation. ChIP Lysis Buffer: 10 mM Tris-HCl pH 8.0, 10 mM NaCl, 0.2 % NP40. ChIP Buffer: 1% Triton X-100, 2 mM EDTA, 150 mM NaCl, 20 mM Tris-HCl pH 8.0. 3C Ligation Buffer: 66 mM Tris-HCl, 5 mM $MgCl_2$, 5 mM DTT, 1 mM ATP, pH 7.5.

**Table S1. Complete list of DNA oligonucleotides used for PCR**.
**Figure S1. RA induction of *CYP26A1* mRNA and recruitment of RARα and phosphorylated RNA polymerase II to the retinoic responsive elements (RARE) and promoter of *CYP26A1* gene.**
**Figure S2. Methylation-demethylation cycles of histone H3K4/K9 induced by RA at *CYP26A1* promoter/RARE and *CASP9* polyA1 and polyA2 chromatin.**
**Figure S3. Recruitment of SUV39H1, H3K9 acetylated, NCoR1 and NCoR2 to the RARE-promoter and polyA addition sites of *CASP9* and *CYP26A1* chromatin.**
**Figure S4. Depletion of the histone demethylases, LSD1 and JMJD2A inhibits RA-induced transcription.**
**Figure S5. Recruitment of LSD1 to the promoter/RARE chromatin of *CYP26A1* is necessary for RA-induced transcription.**
**Figure S6. Expression of the LSD1A mutant inhibits H3K4 and H3K9 methylation cycles at *CASP9* polyA1 and polyA2 chromatin sites.**
**Figure S7. RA induces 8-oxo-dG foci through LSD1.**
**Figure S8. Recruitment of BER and NER enzymes to *CYP26A1* chromatin induced by RA.**
**Figure S9. *CYP26A1* DNA chromatin loops induced by RA.**
**Figure S10. Recruitment of the termination protein Ssu72 to the promoter, RARE, polyA1 and polyA2 of *CASP9* gene.**
**Supplementary Movie. Dynamic chromatin movements triggered by histone demethylation and looping governing transcription induced by retinoids.**

Table S1

ID	PRIMERS FOR mRNA	Locus
mRNA Fw	5'-CCATATGATCGAGGACATCCA-3'	*CASP9*
mRNA Rev	5'-GACTCCCTCGAGTCTCCAGAT-3'	*CASP9*
mRNA Fw	5'-GCAGCCACATCTCTGATCACT-3'	*CYP26A1*
mRNA Rev	5'-TGTTGTCTTGATTGCTCTTGC-3'	*CYP26A1*
mRNA Fw	5'-ATGGAAGGGGTGGAGGAT-3'	*JMJD2A*
mRNA Rev	5'-TTTGTCCCTATTCGGTGCTT-3'	*JMJD2A*
18SF	5'-TCCCCATGAACGAGGAATTC-3'	*h18S*
18SR	5'-GGCCTCACTAAACCATCCAA-3'	*h18S*
mRNA Fw	5'-CGTCATGGTCTTATCAACTTCG-3'	*LSD1*
mRNA Rev	5'-CAGCTACATAGTTTCCTTTGCG-3'	*LSD1*
mRNA Fw	5'-TTTGATGACCCGCAAAGG-3'	*OGG1*
mRNA Rev	5'-AACAACCTTCCCTGTTTCACTT-3'	*OGG1*
mRNA Fw	5'-ACACTCAAGATCTGCTCTTGG-3'	*APE1*
mRNA Rev	5'-TGCCGTAAGAAACTTTGAGTGG-3'	*APE1*
	**PRIMERS FOR ChIP**	**Locus**
ChIP Prom Fw	5'-CGCGCCGCCCCAGAACACG-3'	*CASP9*
ChIP Prom Rv	5'-GCGGGCGGGACGGTAACG-3'	*CASP9*
ChIP RARE Fw	5'-GGAGGCTGAGGCGGGTTTATC-3'	*CASP9*
ChIP RARE Rev	5'-GGTGGAGTGCGGTGGTGTG-3'	*CASP9*
ChIP RARE 5' Fw	5'-TTTTTCTTGCCGCTTTTTCTCATC-3'	*CASP9*
ChIP RARE 5' Rev	5'-TGTCTCAGCCTCCCCAGTAGC-3'	*CASP9*
ChIP PolyA1 Fw	5'-CTAGTAGGCCCCGGTTTGCTGAT-3'	*CASP9*
ChIP PolyA1 Rev	5'-AGTAACGCGTCTTCCTGAGTGGTG-3'	*CASP9*
ChIP PolyA2 Fw	5'-GTTCTCACCCTTGTTGCCTTCCT-3'	*CASP9*
ChIP PolyA2 Rev	5'-TTGCCTATTTCTTCCCTCATTTTG-3'	*CASP9*
Intron 2 Fw/CasF1	5'-GCAAGTACTCAATAATGTTCACC-3'	*CASP9*
Intron 2 Rev/CasE2	5'-TCAACAAAAATTCACCAAAACTCA-3'	*CASP9*
Exon 13 Fw	5'-CCTCCTTGACCAGGCTAATTAC-3'	*TGFBI*
Exon 13 Rev	5'-GGCTGCAACTTGAAGGTTGTG-3'	*TGFBI*
ChIP RARE Fw	5'-GCGGAACAAACGGTTAAAGA-3'	*CYP26A1*
ChIP RARE Rev/C	5'-TGATCGCAGCCAGGAAGA-3'	*CYP26A1*
ChIP PolyA Fw	5'-TCTCAATCCAGAAAGGTTAGCC-3'	*CYP26A1*
ChIP PolyA Rev	5'-ACCTGGTTTCTGCAGTATTGCC-3'	*CYP26A1*
	**PRIMERS FOR 3C**	**Locus**
Cas A	5'-GTCTGTACATGTTCAGTACAATGC-3'	*CASP9*
Cas B	5'-CCACCTGCAGCTCTTCCA-3'	*CASP9*
Cas C	5'-CAGTCATCCGGAGACCTAAACC-3'	*CASP9*
Cas D	5'-AGATGCCCAGCACTATGCTAAG-3'	*CASP9*
Cas E1	5'-GGTGCCTGGCAAATAGCAAT-3'	*CASP9*
Cas E2	5'-TCAACAAAAATTCACCAAAACTCA-3'	*CASP9*
Cas F1	5'-GCAAGTACTCAATAATGTTCACC-3'	*CASP9*
Cas F2	5'-GGGGTGATTCCAGCATAGGTTC-3'	*CASP9*
Cas G	5'-CTTCTGCGTCTGAACTTGAACC-3'	*CASP9*
Cas H	5'-CTGAAAGAAAGAAGGCTGGATGC-3'	*CASP9*
Cas I	5'-AGGAAGGAAACTACCGCTTGCT-3'	*CASP9*
Cas L	5'-TCCCTTTCACCGAAACAGCA-3'	*CASP9*
A	5'-TCACCACTGGACATATTCTTACC-3'	*CYP26A1*
B	5'-GTGGGGTTTGAAGCGCTGG-3'	*CYP26A1*
C	5'-TGATCGCAGCCAGGAAGA-3'	*CYP26A1*
D	5'-TATTTACAGTGGAGCTGGCC-3'	*CYP26A1*
E	5'- CTCAGGTTTGAACATTCAAGC -3'	*CYP26A1*
F	5'-TGTTTGCCAAAGTTTTCAACC-3'	*CYP26A1*

Supplementary Table S1. Complete list of DNA oligonucleotides used for PCR. On the left is shown the primer identification tag (ID); on the center, the DNA sequence and the specific experiments; on the right, the genes or loci corresponding to the specific primers.

Fig. S1

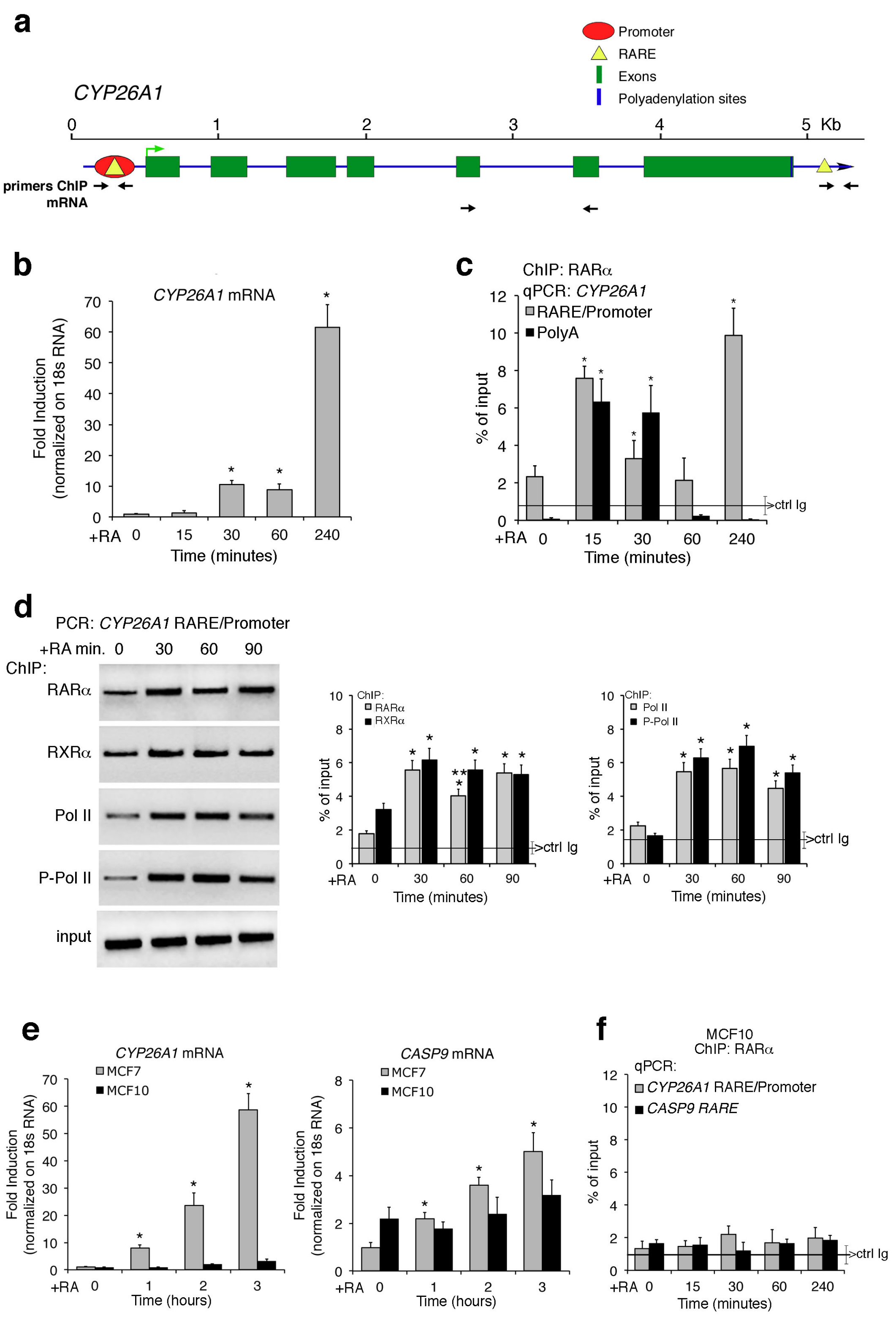

Fig S1. RA induction of *CYP26A1* mRNA and recruitment of RARα and phosphorylated RNA polymerase II to the retinoic responsive elements (RARE) and promoter of *CYP26A1* gene.

a. Structure of *CYP26A1* gene. The transcription start site and the direction of transcription are indicated by a green arrow; the exons, promoter, polyA addition sites and RARE are shown by different colors indicated at the upper right corner. The black arrows indicate the primers used for ChIP and mRNA analysis. **b.** Total RNA was prepared from MCF-7 cells as described in Materials and Methods, exposed to 300 nM retinoic acid (RA) for 15, 30, 60 and 240 minutes and analyzed by qPCR with specific primers (panel **a**) to *CYP26A1* mRNA normalized to 18s RNA levels. The statistical analysis derives from at least 3 experiments in triplicate (n ≥9); *p <0.01 (matched pairs *t* test) compared to RA-un-stimulated sample. **c.** qChip analysis of RA-induced occupancy of RARα at the promoter/RARE of *CYP26A1* gene. MCF7 cells were stimulated with 300 nM RA for 15, 30, 60 and 240 minutes. The chromatin was immunoprecipitated with antibodies directed against RARα. **d.** ChIP analysis of RA-induced occupancy of RARα, RXRα, RNA polymerase II (Pol II) and phosphorylated RNA polymerase II (p-Pol II) (at the promoter/RARE of *CYP26A1* gene. MCF7 cells were stimulated with 300 nM RA for 30, 60 and 90 minutes. The chromatin was immunoprecipitated with antibodies directed against RARα, RXRα, Pol II and p-Pol II. The panels show a semiquantitative (left panel) or a qPCR (center and right panels) assay of the ChIP reactions. The black, horizontal, line in each plot indicates the percent of input from a control ChIP (Ab: non immune serum, brackets $\pm$SD). The statistical analysis derives from at least 3 experiments in triplicate (n=≥9); *p <0.01 (matched pairs *t* test) compared to RA-un-stimulated sample; **p<0.01 (matched pairs *t* test) comparing 30 to 60 min.

Fig. S2

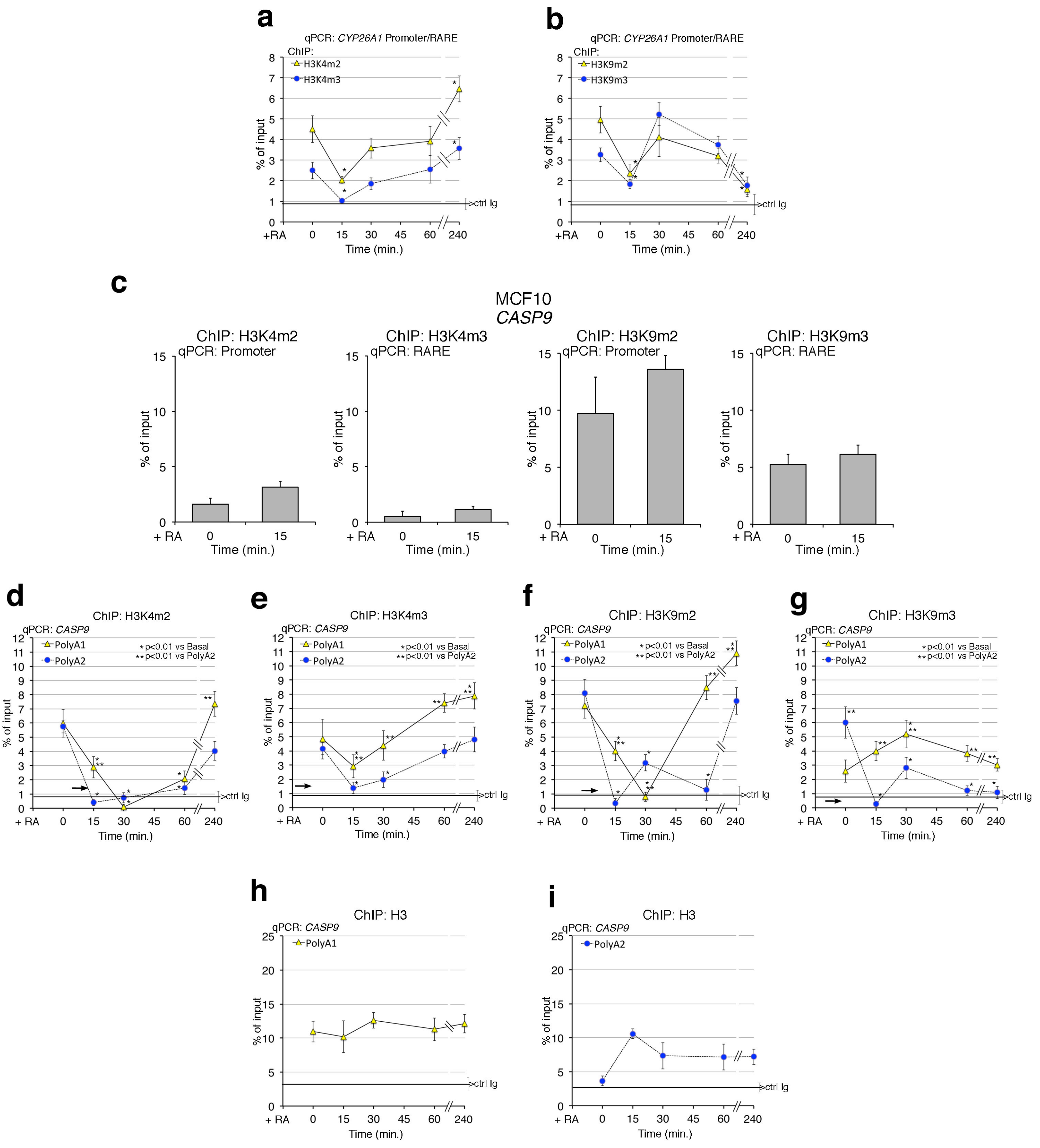

Fig S2. Methylation-demethylation cycles of histone H3K4/K9 induced by RA at *CYP26A1* promoter/RARE and *CASP9* polyA1 and polyA2 chromatin.

MCF7 cells were serum starved and exposed to 300 nM RA at the indicated times (15, 30, 60 and 240 min). qChIP was carried out using specific antibodies recognizing H3K4me3, H3K4me2, H3K9me3 and H3K9me2. **a, b.** H3K4 and H3K9 me2 and me3 occupancy at the *CYP26A1* promoter and RARE chromatin. **c.** H3K4 and H3K9 me2/3 occupancy at the *CASP9* promoter and RARE chromatin in MCF10 cells. **d, e,** H3K4me2 and H3K4me3 occupancy at polyA1 and polyA2 addition sites of *CASP9* gene. **f, g,** H3K9me2 and H3K9me3 occupancy at polyA1 and polyA2 addition sites of *CASP9* gene. **h, i.** ChIP histone H3 at polyA1 and polyA2 after RA induction. The black, horizontal, line in each plot indicates the percent of input from a control ChIP (Ab: non immune serum, brackets $\pm$SD). The statistical analysis derives from at least 3 experiments in triplicate ($n \geq 9$); *p <0.01 (matched pairs *t* test): compared to the RA-un-stimulated sample; **p<0.01 (student *t* test): comparison between each region at same RA time.

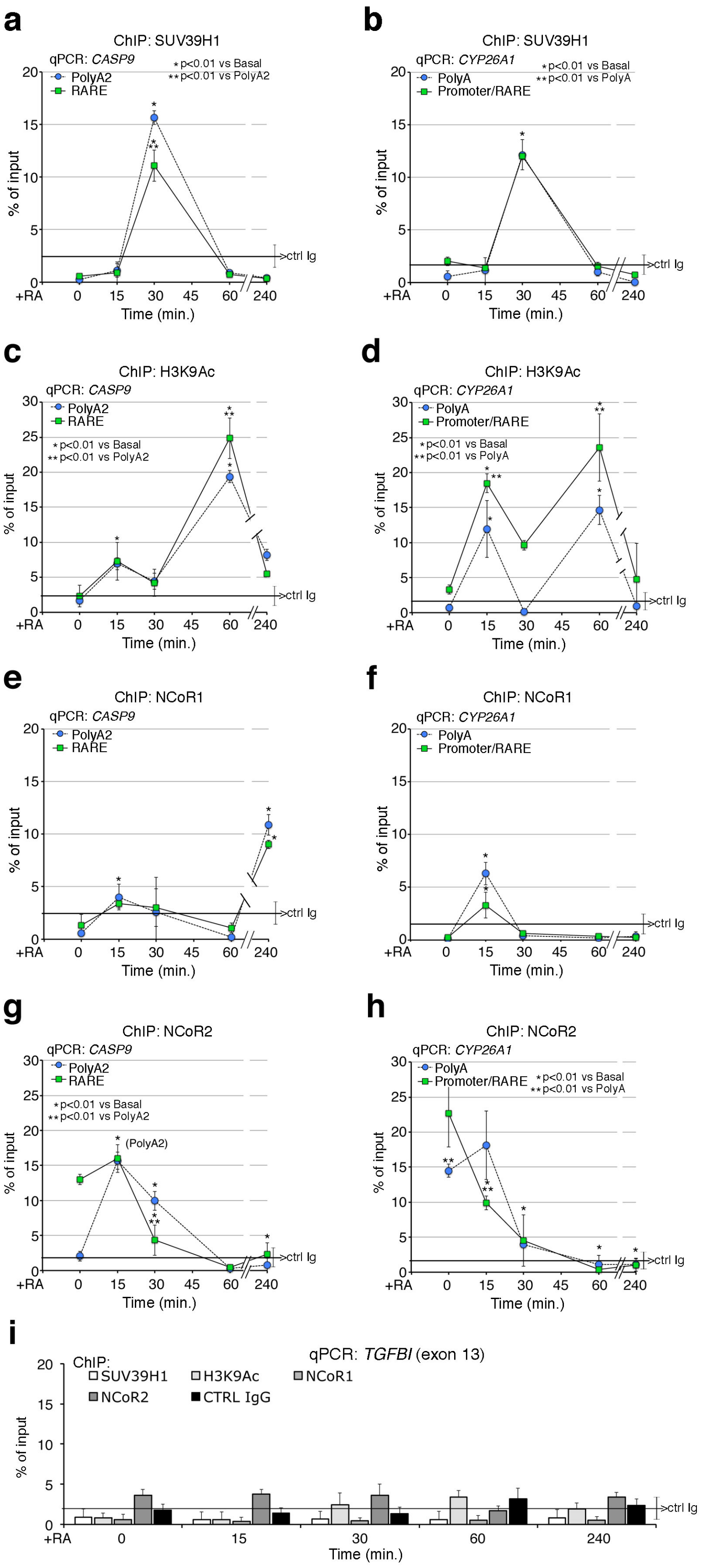

Fig. S3. Recruitment of SUV39H1, H3K9 acetylated, NCoR1 and NCoR2 to the RARE-promoter and polyA addition sites of *CASP9* and *CYP26A1* chromatin

MCF7 cells were exposed to RA for the periods indicated and analyzed by ChIP with specific antibodies indicated at the top of each panel. The regions of the chromatin analyzed are indicated at the upper left of each panel and were amplified from the same immunoprecipitate. The statistical analysis derives from at least 3 experiments in triplicate (n≥9); *p <0.01 (matched pairs *t* test): compared to the RA-un-stimulated sample; **p<0.01 (student *t* test): comparison between each region at same time. **i.** a control gene non-RA-responsive (*TGFBI,* exon 13) was included in the analysis. The black, horizontal, line in each plot indicates the percent of input from a control ChIP (Ab: non immune serum, brackets ±SD).

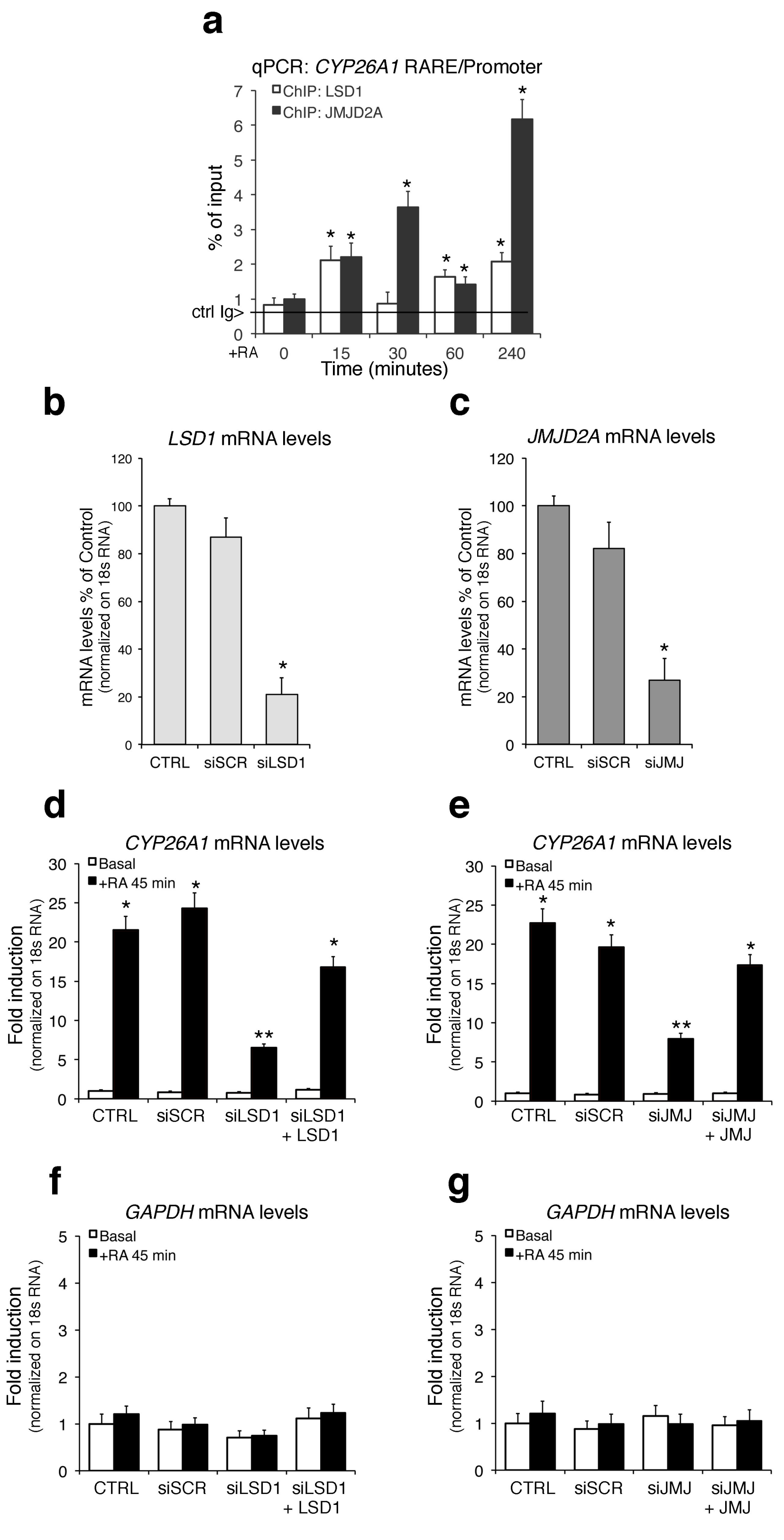

Fig S4. Depletion of the histone demethylases, LSD1 and JMJD2A inhibits RA-induced transcription.

a. Recruitment of LSD1 and JMJD2A to the promoter/RARE region of *CYP26A1* gene. MCF7 cells were serum starved and exposed to 300 nM RA at the indicated times (15, 30, 60 and 240 min). qChIP was carried out using specific antibodies recognizing LSD1 and JMJD2A. The panel shows the time course of the recruitment of LSD1 (white) and JMJD2A (black) to the promoter/RARE chromatin of *CYP26A1* by qPCR. **b, c, d, e, f, g.** MCF7 were transiently transfected with LSD1 siRNA or JMJD2A siRNA or scrambled (siSCR) with or without the wild type gene expressing vectors. The efficiency of the treatments was measured by qPCR using primers for *LSD1* and *JMJD2A* mRNAs (panels **b** and **c**). Transfection efficiency was monitored by FACS (Alexa Fluor or co-transfected pEGFP Vector). After 48 h, total RNA was prepared from control cells (starved) or RA induced cells (300 nM RA for 45 min) and analyzed by qPCR with specific primers to *CYP26A1* mRNA (panels **d** and **e**) and *GAPDH* mRNA (panels **f** and **g**). The statistical analysis derives from at least 3 experiments in triplicate(n=≥9); *p <0.01 (matched pairs *t* test) compared to RA-unstimulated sample; **p<0.01 (student *t* test): comparison between siSCR and specific siRNA.

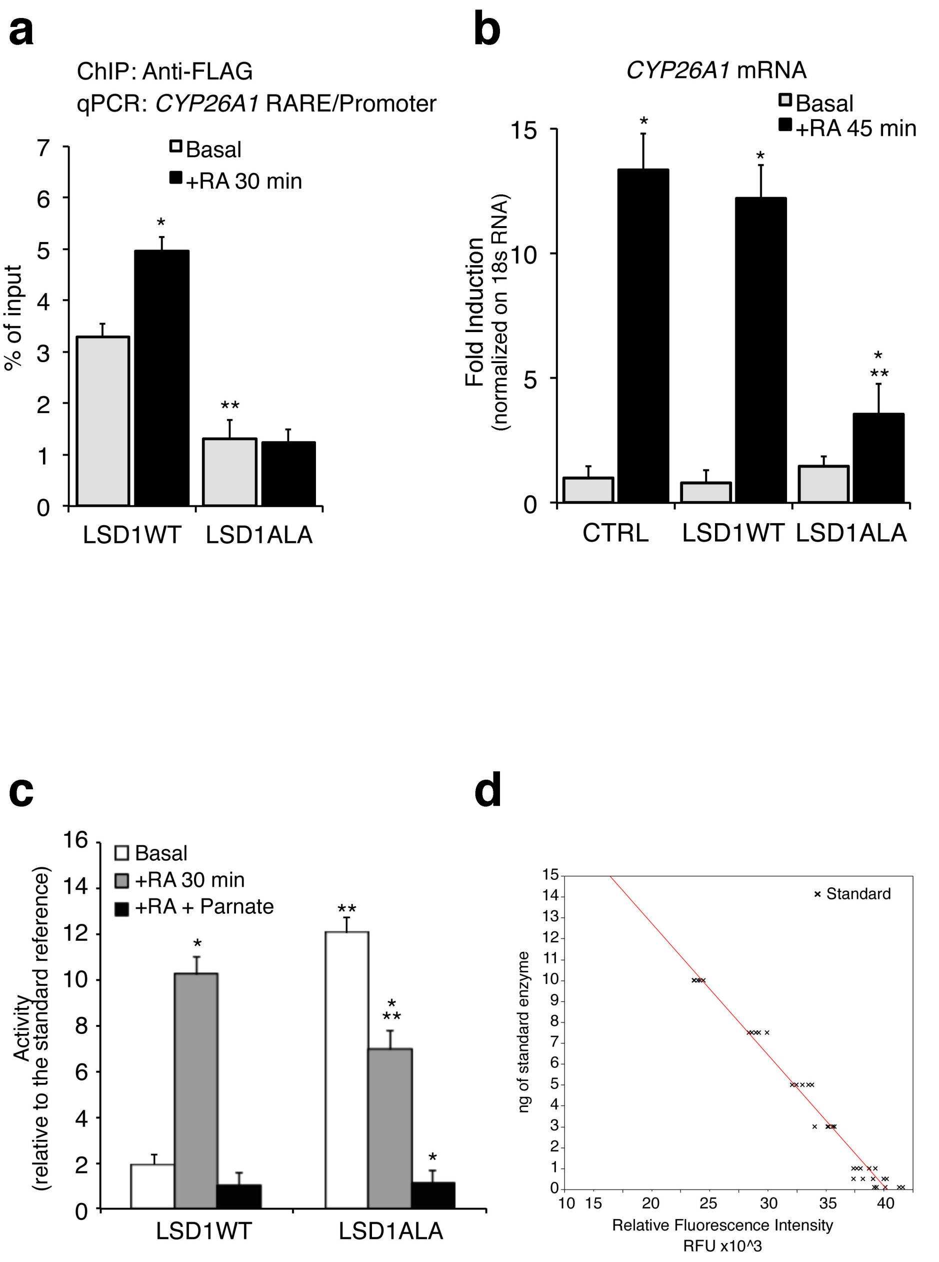

Figure S5. Recruitment of LSD1 to the promoter/RARE chromatin of *CYP26A1* is necessary for RA-induced transcription.

a. Recruitment of LSD1 mutant to the promoter/RARE of *CYP26A1* chromatin. Cells transfected with the LSD1WT-FLAG or the LSD1ALA-FLAG mutant (see Materials and Methods), starved (basal) or treated with 300 nM RA for 30 minutes, were analyzed by ChIP with anti-FLAG antibodies as described in Material and Methods. **b.** RA induction of *CYP26A1* expression in cells transfected with LSD1ALA mutant. Cells were transfected with the LSD1WT-FLAG or the LSD1ALA-FLAG mutant and 48 h later stimulated 45 min with RA. PCR was carried out with specific *CYP26A1* primers as indicated (Material and Methods). **c.** Activity of the LSD1ALA mutant. Cells transfected with LSD1WT-FLAG or the LSD1ALA-FLAG mutant were exposed to RA for 30 min in the presence or absence of parnate (tranylcypromine) (1 μM), a monoamino-oxidase inhibitor. Total cell extracts were prepared and analyzed for LSD1 activity with a fluorescent H3K4me2 substrate (EpiQuik™ Histone Demethylase Activity/Inhibition Assay Kit). **d.** A standard curve was generated by serially diluting the purified enzyme. The statistical analysis derives from at least 3 experiments in triplicate(n=≥9); *p <0.01 (matched pairs *t* test) compared to RA-unstimulated sample; **p<0.01 (student *t* test): comparison between LSD1WT and LSD1ALA.

Fig. S6

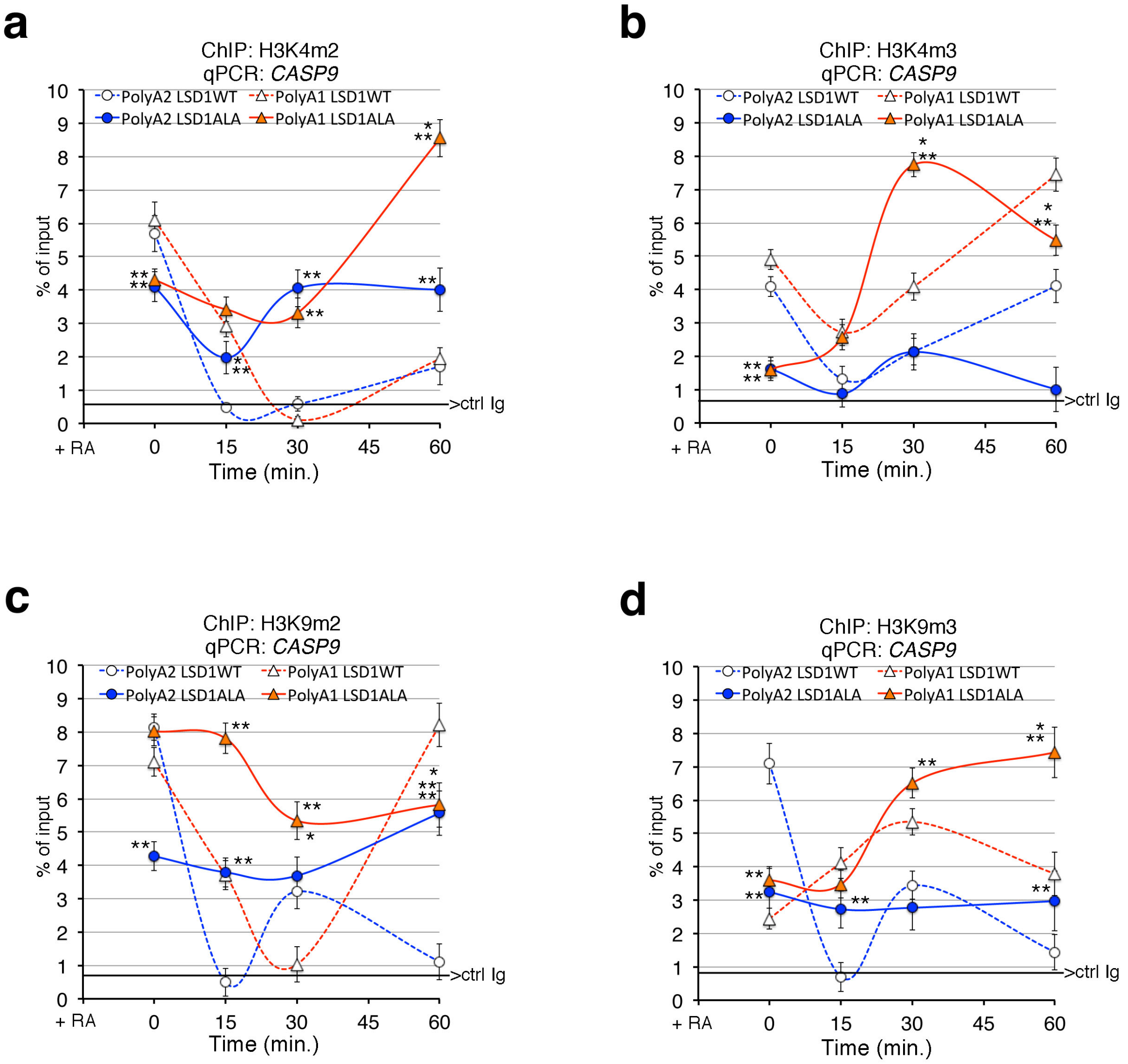

Figure S6. Expression of the LSD1A mutant inhibits H3K4 and H3K9 methylation cycles at *CASP9* polyA1 and polyA2 chromatin sites.
Cells transfected with the LSD1WT-FLAG or the LSD1ALA-FLAG mutant were analyzed 36 h later by ChIP with specific antibodies to H3K4me2 (**a**), H3K4me3 (**b**) and H3K9me2 (**c**) and H3K9me3 (**d**). The statistical analysis derives from at least 3 experiments in triplicate ($n \geq 9$); *p <0.01 (matched pairs *t* test): compared to the RA-unstimulated sample; **p<0.01 (student *t* test): comparison between control and the LSD1ALA expressing cells.

Fig. S7

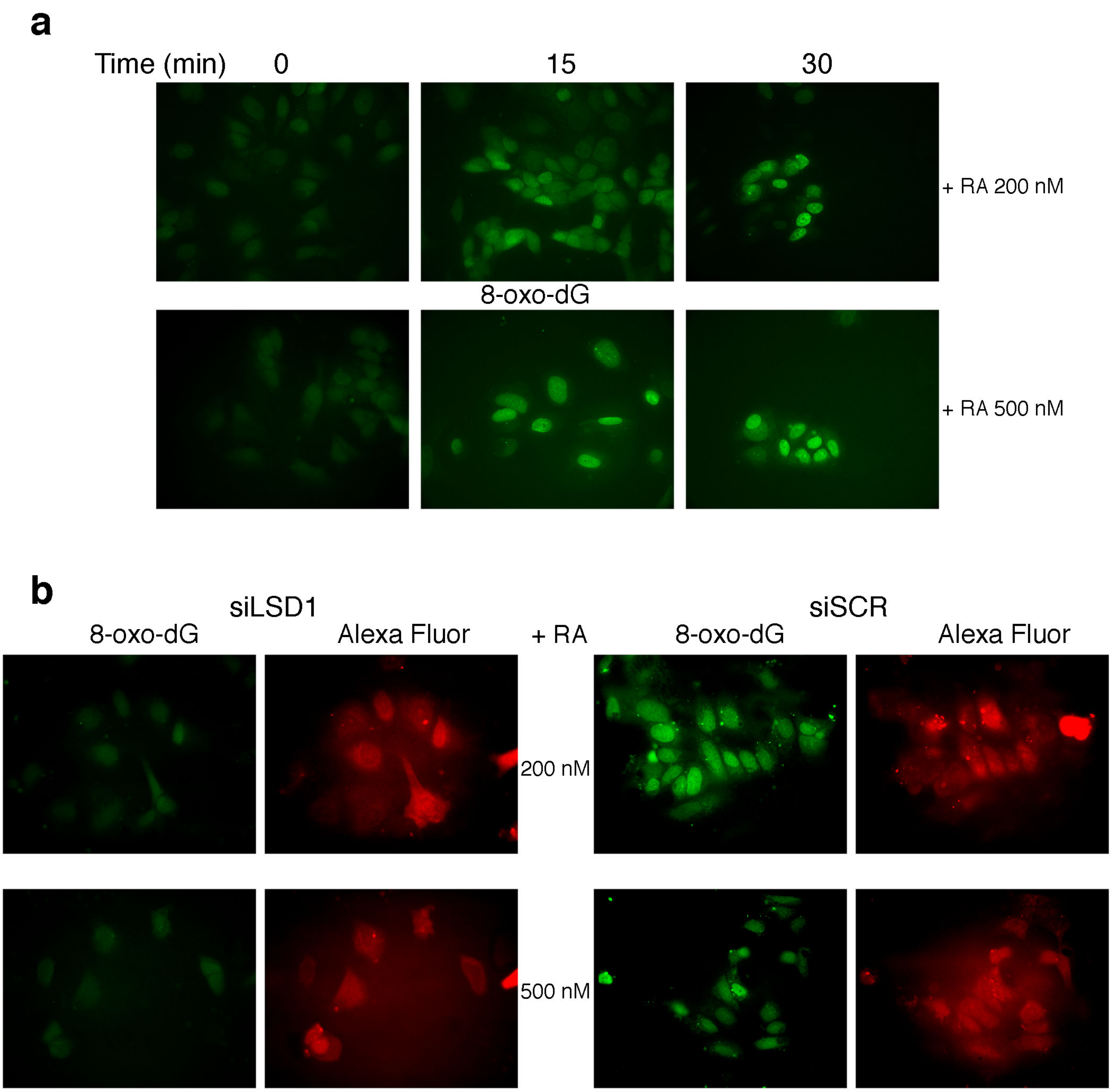

Fig S7. RA induces 8-oxo-dG foci through LSD1.

a. Time course of 8-oxo-dG staining in cells exposed to RA. MCF7 cells seeded onto glass slides, were starved and treated with 200 nM or 500 nM RA for 15 and 30 min, fixed, and analyzed for the presence of 8-oxo-dG as described in Material and Methods. The 8-oxo-dG signal (green fluorescence) was quantified by ImageJ 1.43 (NIH). Positive cells, containing a signal 2 S.D. higher than controls (CTRL), were 42 and 57% after 15 minutes and 67 and 72 % after 30 minutes of 200 or 500 nM of RA, respectively. **b.** LSD1 knockdown inhibits RA-induced 8-oxo-dG accumulation. Cells were transfected with siRNA targeting LSD1 as described in Material and Methods. 24 h later the cells were exposed to 200 or 500 nM RA for 30 minutes and 8-oxo-dG foci monitored as described above (right panels). Transfection efficiency was monitored using Alexa Fluor Red Fluorescent Control.

Fig. S8

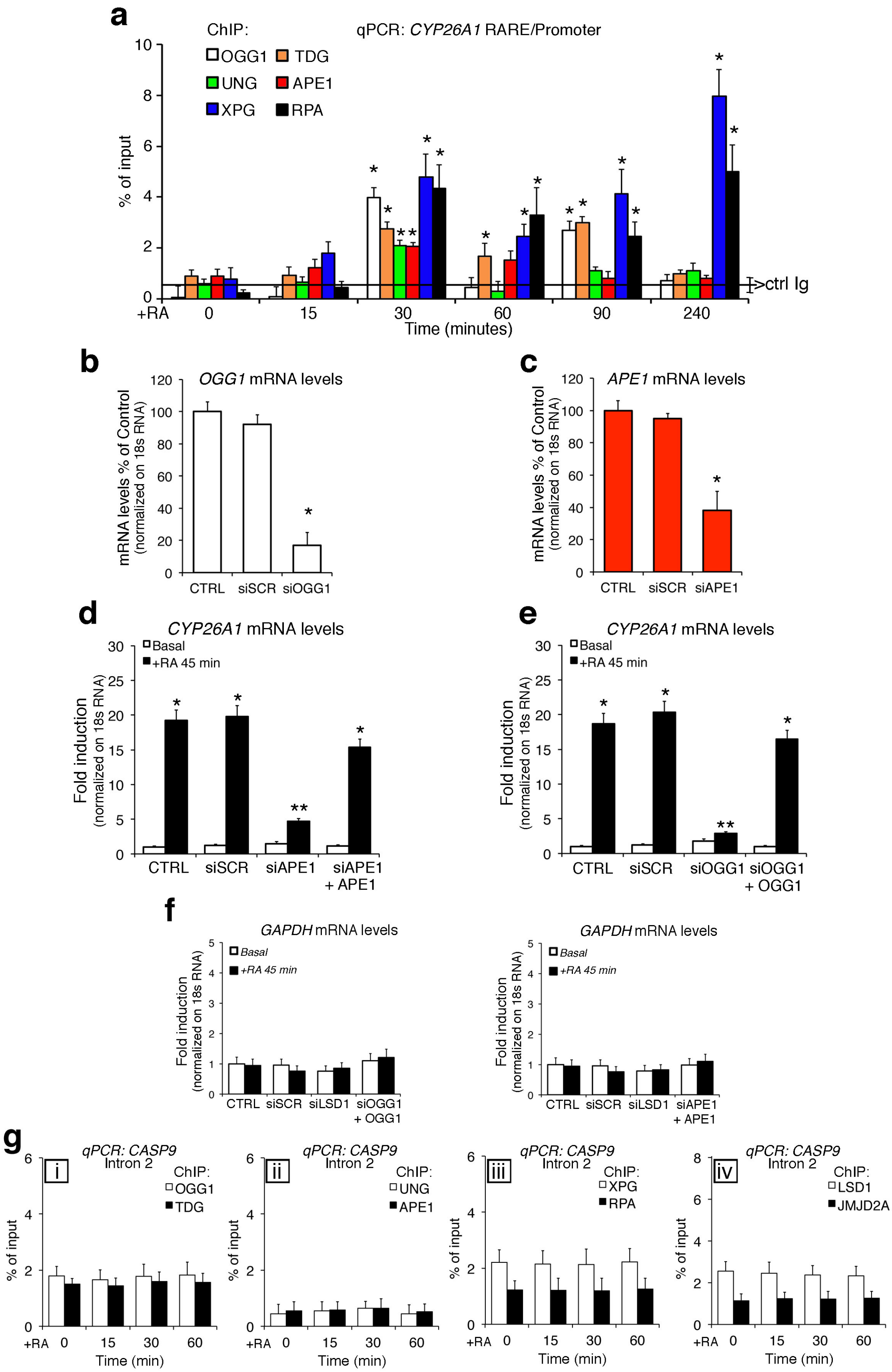

Figure S8. Recruitment of BER and NER enzymes to *CYP26A1* chromatin induced by RA.

a. MCF7 cells, starved or treated with RA for 15, 30 and 240 min, were analyzed by qChIP using specific antibodies recognizing the 8-oxoguanine–DNA glycosylase-1 (OGG1), AP endonuclease (APE1), Thymine-DNA glycosylase (TDG), Uracil-DNA glycosylase (UNG), XPG and RPA. The black, horizontal, line indicates the percent of input from a control ChIP (Ab: non immune serum). **b, c, d, e, f**. Depletion of OGG1 and APE1 inhibits RA-induced transcription. Panels **b** and **c** show the levels of O*GG1* and *APE1* mRNAs in cells exposed to specific targeting siRNAs, respectively. Panels **d** and e show RA-induction of *CYP26A1* mRNA in OGG1 and APE1-depleted cells, respectively. Panel f shows the GAPDH mRNA levels in OGG1 and APE1-depleted cells. **g** shows the ChIP analysis of *CASP9*, II intron, in cells exposed to RA for various periods with specific antibodies to OGG1, TDG (i); UNG, APE1 (ii); XPG, RPA (iii); LSD1, JMJD2A (iv). The statistical analysis derives from at least 3 experiments in triplicate (n≥9); *p <0.01 (matched pairs *t* test) compared to RA-unstimulated sample; **p<0.01 (student *t* test): comparison between siSCR and specific siRNA.

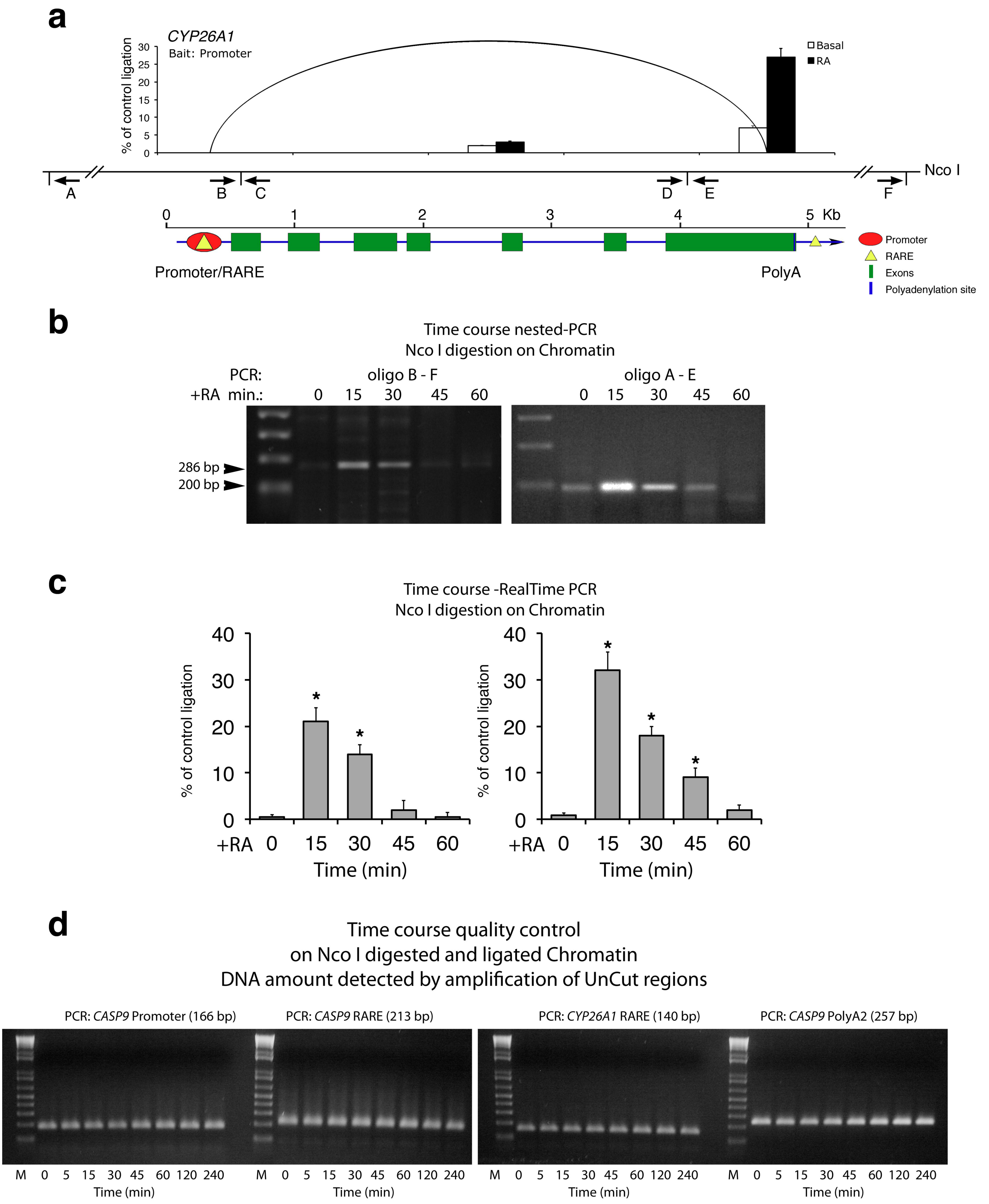

Figure S9. *CYP26A1* DNA chromatin loops induced by RA.

a. Schematic diagram of *CYP26A1* gene regions. The curved lines indicate the 5' and 3' of the loops detected by 3C technique. The histograms show the frequency of the ligated fragments compared to the ligation of the same cloned fragments from genomic DNA. The primers are indicated by arrows. **b.** Time course of loop formation following RA treatment. Semiquantitative nested PCR after digestion with NcoI and ligation of chromatin of cells exposed to RA for various periods of time. The specific products detected with forward B-F and reverse A-E primers are indicated by arrows. **c.** qPCR analysis of the 3C products in chromatin of cells exposed to RA for various periods of time. The results shown derive from a least 3 experiments in triplicate ($n \geq 9$). *$p<0.01$ as compared to untreated samples. **d.** Time course of DNA detected by amplification of UnCut regions in NcoI digested and ligated chromatin.

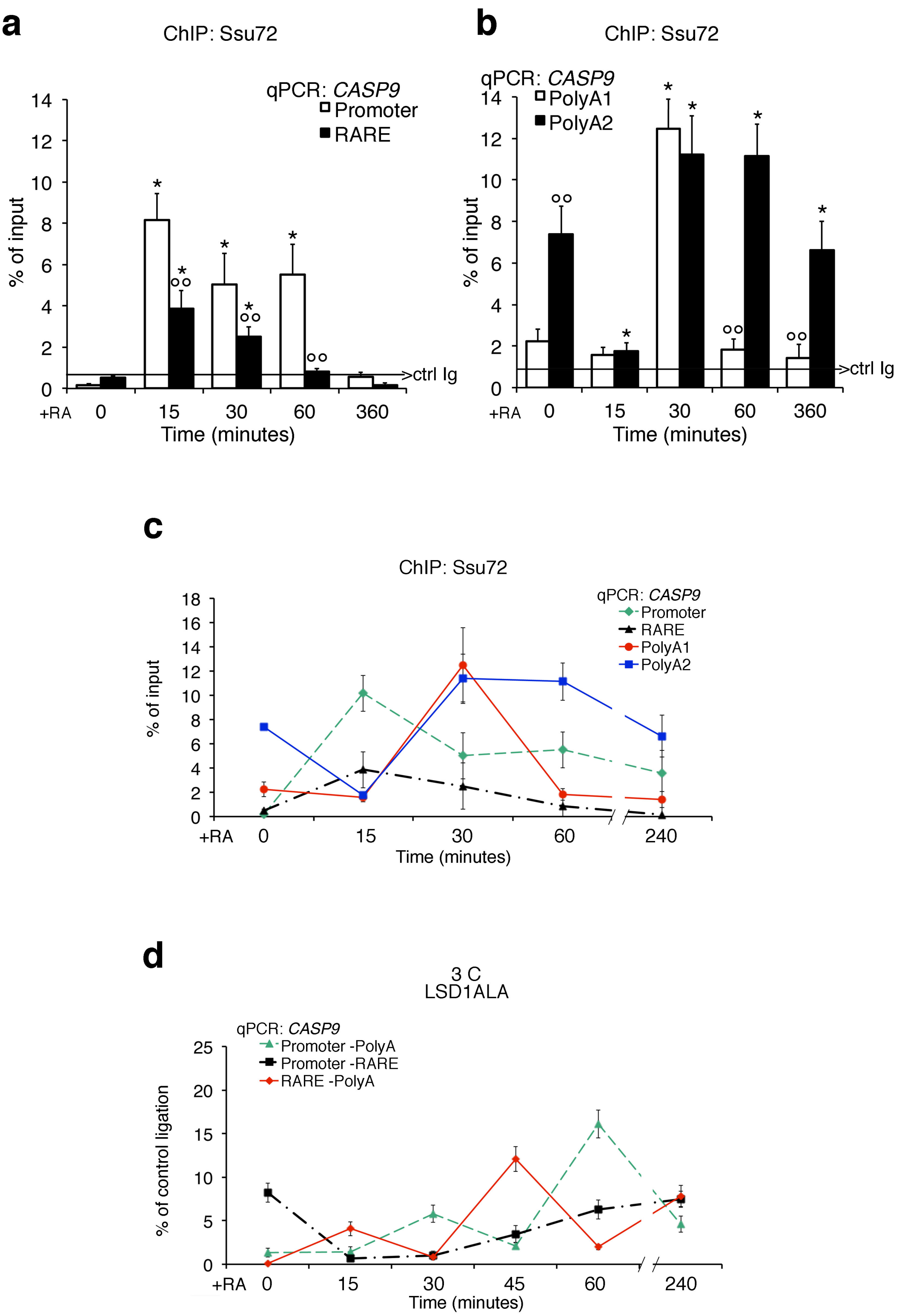

Figure S10. Recruitment of the termination protein Ssu72 to the promoter, RARE, polyA1 and polyA2 of *CASP9* gene.

Cells were exposed to RA for various periods of time and subjected to ChIP analysis with specific antibodies to Ssu72 protein. **a, b** indicate the fraction of Ssu72 bound to the promoter and RARE or to the polyA1 and polyA2 of *CASP9* gene, respectively. **c.** The panel shows the time course of Ssu72 recruitment. The statistical analysis derives from at least 3 experiments in triplicate (n≥9); *p <0.01 (matched pairs *t* test): compared to the RA-unstimulated sample; °°p<0.01 (student *t* test): comparison between two amplicons. **d.** 3C analysis of *CASP9* chromatin in MCF7 cells transfected with the LSD1ALA mutant and exposed to 300 nM of RA for various periods of time; the panel shows the time course of loop formation; data were collected from Real Time qPCR and from semi-quantitative, nested PCR experiments.
